# Covalent targeting of non-cysteine residues in PI4KIIIβ†

**DOI:** 10.1039/d3cb00142c

**Published:** 2023-10-17

**Authors:** Brett Cosgrove, Emma K. Grant, Sophie Bertrand, Kenneth D. Down, Don O. Somers, John P. Evans, Nicholas C. O. Tomkinson, Michael D. Barker

**Affiliations:** a Medicinal Chemistry, GlaxoSmithKline Medicines Research Centre Stevenage SG1 2NY UK; b Department of Pure and Applied Chemistry, University of Strathclyde Glasgow G1 1XL UK Nicholas.Tomkinson@strath.ac.uk; c Structural and Biophysical Science, GlaxoSmithKline Medicines Research Centre Stevenage SG1 2NY UK; d Screening, Profiling and Mechanistic Biology, GlaxoSmithKline Medicines Research Centre Stevenage SG1 2NY UK

## Abstract

The synthesis and characterisation of fluorosulfate covalent inhibitors of the lipid kinase PI4KIIIβ is described. The conserved lysine residue located within the ATP binding site was targeted, and optimised compounds based upon reversible inhibitors with good activity and physicochemical profile showed strong reversible interactions and slow onset times for the covalent inhibition, resulting in an excellent selectivity profile for the lipid kinase target. X-Ray crystallography demonstrated a distal tyrosine residue could also be targeted using a fluorosulfate strategy. Combination of this knowledge showed that a dual covalent inhibitor could be developed which reveals potential in addressing the challenges associated with drug resistant mutations.

## Abstract 2

The synthesis and characterisation of fluorosulfate covalent inhibitors of the catalytic lysine, a distal tyrosine or both residues in the lipid kinase PI4KIIIβ is described.

## Graphical abstract

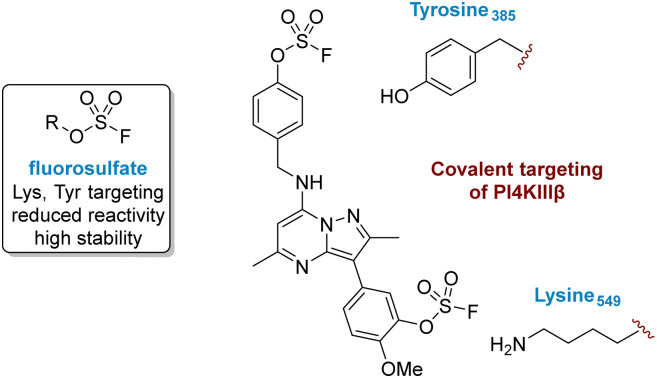

## Introduction

The World Health Organisation has predicted that Chronic Obstructive Pulmonary Disease (COPD) will become the third leading cause of death worldwide by 2030.^1^ Exacerbations of COPD are frequently driven by viral infections, especially human rhinovirus (HRV).^2,3^ Strong evidence has implicated the lipid kinase PI4KIIIβ, a phosphatidylinositol kinase widely expressed in mammalian cells, in the replication of several RNA viruses, including HRV, due to its signalling pathway.^4^ As a result, PI4KIIIβ is an appealing target for prevention of HRV-driven exacerbations in respiratory diseases. PI4KIIIβ has a long re-synthesis rate in human T-cells, which suggests small molecule inhibitors with long residency time in the active site of this protein could be a viable approach in this therapeutic area. Inspired by the renaissance of covalent inhibition as a mechanism to target proteins of interest,^5^ this strategy was chosen for the development of inhibitors of PI4KIIIβ with a prolonged duration of action.

**Fig. 1:**
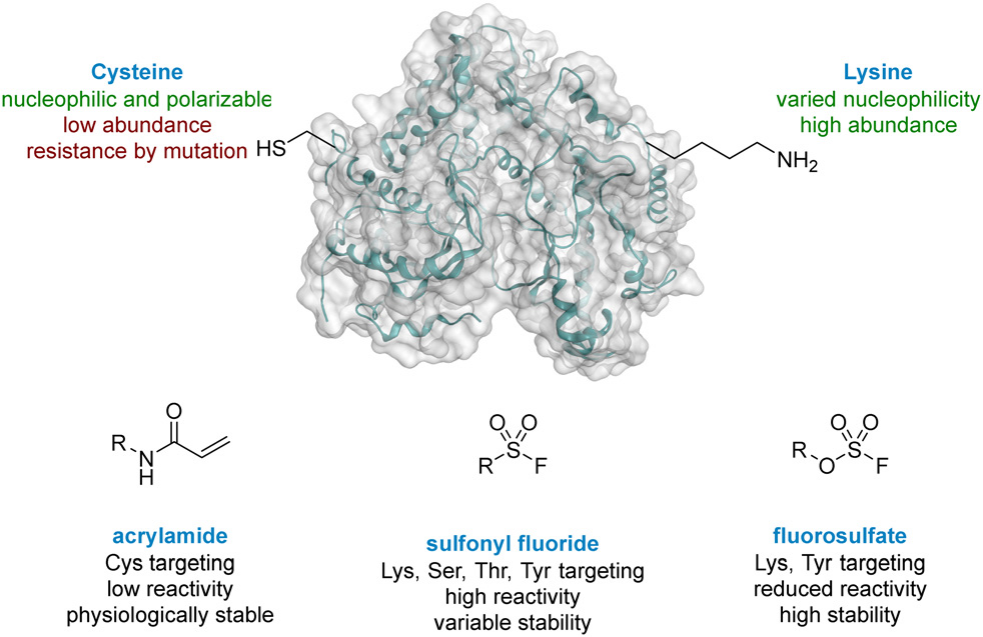
Comparison of cysteine and lysine residues as targets in covalent drug discovery.

Cysteine targeted covalent inhibition has been a focus of discovery science for many years, although concerns around specificity remain.^6,7^ Targeting the thiol of cysteine, a nucleophilic and polarizable functionality, can permit the use of low reactivity warheads to minimise off-target effects and non-specific binding,^8,9^ which has led to the breakthrough discovery of compounds such as Afatinib and Ibrutinib for the treatment of chronic diseases in oncology.^10,11^ Both these drugs target a cysteine residue *via* a Michael addition to an α,β-unsaturated amide (Fig. 1).^12,13^ Despite these successes, two significant limitations associated with cysteine targeted covalent drug design have been identified.^14^ (I) The natural abundance of cysteine is low and many cysteine residues are engaged in disulfide bonds, further decreasing the availability of this functionality.^15^ (II) Resistance can develop in a clinical setting,^16,17^ where the cysteine residue can undergo single point mutation to methionine or serine, preventing formation of a covalent adduct when dosed with the drug. As a result, there is a need to target alternative nucleophilic amino acids using different covalent warheads.

Lysine is one of the most prevalent naturally occurring amino acids, with an estimated 650 000 residues present in the human proteome, and has recently generated interest in covalent drug design.^14,18^ Despite its prevalence this critical amino acid residue remains underexplored, probably due to its protonation under physiological conditions, rendering the amine unreactive to electrophiles.^19^ The nucleophilicity of amino acid residues can be modulated significantly by the local protein microenvironment.^20–22^ Interestingly, perturbation of lysine p*K*_a_ can be an advantage in covalent drug design through the provision of selectivity to more nucleophilic residues.^23^ Pioneering work of Cravatt identified several hundred lysine residues with heightened reactivity, which could, in principal, be targeted by electrophilic small molecules.^24^ In addition, computational methods for predicting reactive residues in kinases are actively being developed^25^ although challenges remain.^26,27^

Due to their potential, reactive warheads targeting lysine residues are emerging as effective tools in covalent drug discovery.^28^ For example, sulfonyl fluorides (Fig. 1) have been shown to react with a variety of amino acid side-chains including lysine.^29,30^ Taunton reported a sulfonyl fluoride covalent warhead to probe the kinome, identifying a compound that formed covalent adducts with 133 different endogenous kinases.^31^ Despite their promising reactivity profile, there are stability issues associated with sulfonyl fluorides that may hinder their applicability.^32,33^ Increased stability can be obtained with fluorosulfates,^34,35^ and new synthetic methods to access this functionality,^36,37^ coupled with their tempered reactivity in comparison to other sulfur(vi) electrophiles, render them an attractive class of warhead.^38,39^

Campos and co-workers reported that a nucleophilic lysine residue in PI3Kδ could be covalently modified using activated phenolic esters of the lipid kinase PI3Kδ^26^ where they showed that selectivity in the formation of the initial enzyme–inhibitor complex was critical for achieving potent and selective covalent inhibition. Based on this insight we embarked on an investigating to covalently target the conserved lysine residue in PI4KIIIβ as a general approach for lipid kinase drug design.

## Results and discussion

**Fig. 2:**
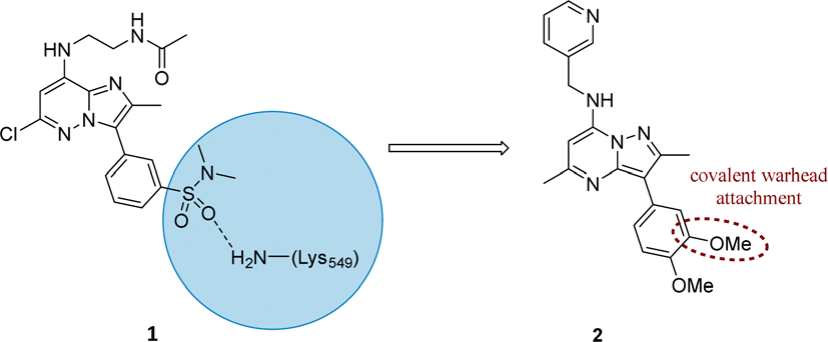
Design strategy for the development of a covalent inhibitor for PI4KIIIβ.

Literature describing PI4KIIIβ has highlighted a hydrogen bonding interaction between the sulfonamide moiety in 1 and Lys_549_ in the ATP binding pocket of the protein (Fig. 2).^40^ Drawing inspiration from this finding, compound 2 was selected as a suitable starting point for this work due to its activity against the protein of interest alongside the excellent physicochemical profile displayed by this ligand.^41^

Surprisingly, a sulfonyl fluoride on this scaffold proved susceptible to rapid hydrolysis (see ESI† for further details), therefore, arylfluorosulfates were examined as the covalent warhead due to their reduced electrophilicity and increased stability.^33,35^ Initially, two fluorosulfate compounds 3 and 4 were prepared and screened against PI4KIIIβ in both a biochemical- and cell-based assay (Table 1). Covalent modification of a protein is driven by a two-step process.^42^ Reversible binding of the inhibitor within the active site places the electrophilic moiety in close proximity to the nucleophilic residue being targeted. Upon formation of this complex, the covalent adduct can then be formed. Formation of the reversible complex is time dependent and is frequently rate limiting.^5^ In our enzyme assay, a 40 minutes pre-incubation was used compared to a 48 hours incubation for the cellular cytopathic effect (CPE) assay where the prolonged incubation may be necessary for the inhibitors to covalently modify the protein (see ESI† for full details). Within the cell assay, high levels of intracellular ATP (10 mM) were present which can outcompete reversible competitive inhibitors. Therefore, the measured pIC_50_ values were likely to drop from the enzyme to the cell assay for reversible inhibitors, whereas for covalent inhibition, the potency could be expected to translate or even increase.

**Table 1:** Biochemical and physicochemical data for compounds 3–6

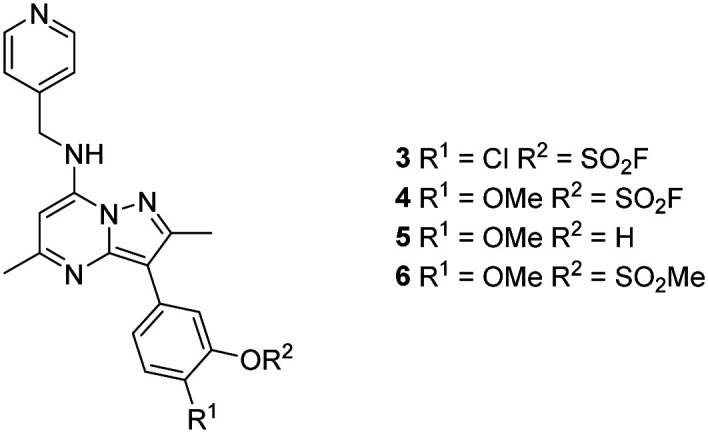						
	PI4KIIIβ pIC_50_^a^	CPE pIC_50_^b^	Δ(CPE-PI4KIIIβ)	Solubility (μg mL^−1^)	Chrom LogD_7.4_	AMP (nm s^−1^)
3	6.3 ± 0.060	6.8 ± 0.063	0.5	12	7.2	n.d.
4	7.2 ± 0.101	8.2 ± 0.058	1.0	15	6.2	215
5	7.2 ± 0.145	7.0 ± 0.063	−0.9	—	—	510
6	8.1	7.6 ± 0.005	−0.5	—	—	360

a Compound incubated with protein for 40 minutes.

b Compound incubated with cells for 48 hours.

An increase in potency was observed for the fluorosulfate containing compounds 3 and 4 from the enzyme to the cell assay, indicating a potential time dependency associated with the activity for these compounds (Table 1). Whilst the phenol control 5 was more potent than compound 4 in the enzyme assay it was a log unit less potent in the CPE assay. Both compounds 4 and 5 were permeable which eliminates the possibility of 5 not being able to enter the cell. This suggested that the increase in activity in the cell assay for compound 4 was not driven by hydrolysis of the fluorosulfate group to the phenol. Although the physiochemical properties were not improved, the increase in activity within the cell assay was encouraging. Compound 4 was below 10 nM activity in the CPE assay and became the lead compound for characterising a potential covalent adduct.

**Fig. 3:**
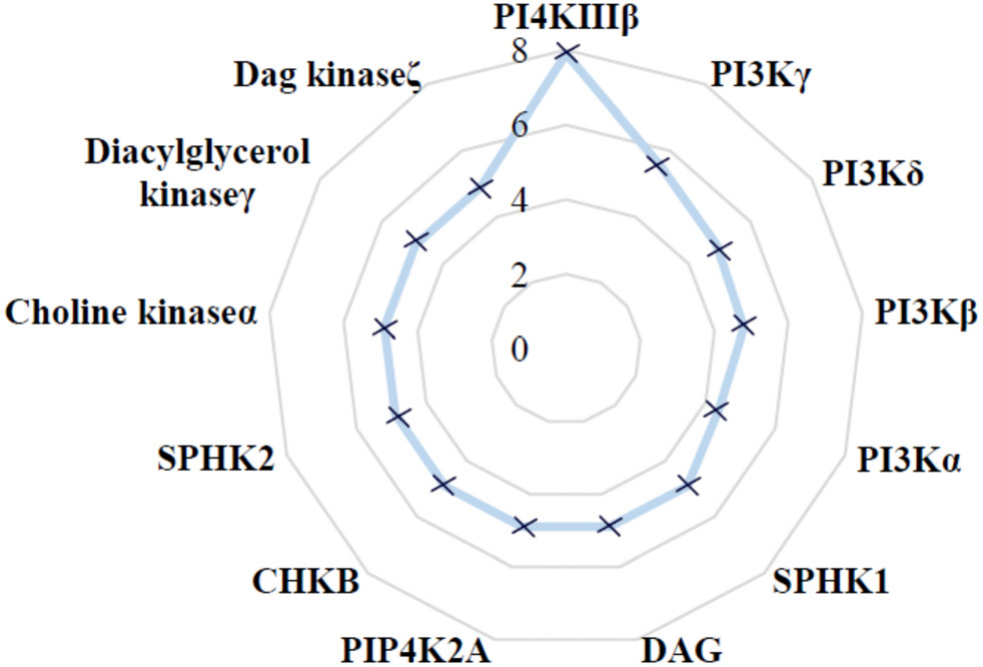
Lipid kinase pIC_50_ screening data for 4.

The selectivity profile of compound 4 over other lipid kinases was established (Fig. 3). Pleasingly, >100-fold selectivity against other lipid kinases was observed. PI3Kγ had a pIC_50_ of 5.5, whilst all other lipid kinases were inactive at the concentrations tested (pIC_50_ < 5).

**Fig. 4:**
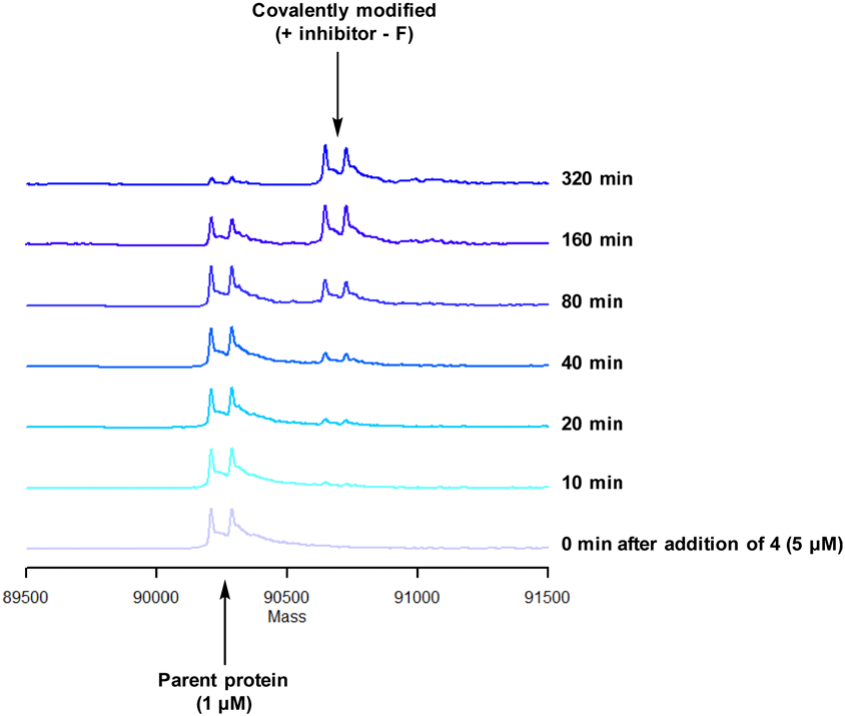
Time course of compound 4 reacting with recombinant PI4KIIIβ.

To characterise the interaction of 4 and PI4KIIIβ, mass spectrometry was used to probe covalent modification of the protein. Concentration and time dependant mass spectrometry experiments are useful tools for determining the selectivity of covalent modification and provide an indication of the kinetics of the process.^43^ Multiple covalent adducts are indicative of nonspecific binding and promiscuous reactivity which represents a major challenge of non-reversible inhibition in drug discovery. In addition, the time taken to label a protein provides insight on the mechanism of modification. A time course mass spectrometry experiment was carried out for compound 4 (5 μM) with PI4KIIIβ (1 μM) (Fig. 4).

Pleasingly, compound 4 covalently modified recombinant PI4KIIIβ protein (Fig. 4). The covalent modification took around 2 h to reach 50% conversion with 5 eq. of 4 under neutral conditions. The parent protein displayed two peaks by mass spectrometry corresponding to a phosphorylated and non-phosphorylated form in roughly equal proportions. The mass shift after covalent labelling was consistent with addition of 4 minus the fluoride ion, correlating with attack of a nucleophilic residue onto the electrophilic sulfur atom and expelling a fluoride leaving group.

**Fig. 5:**
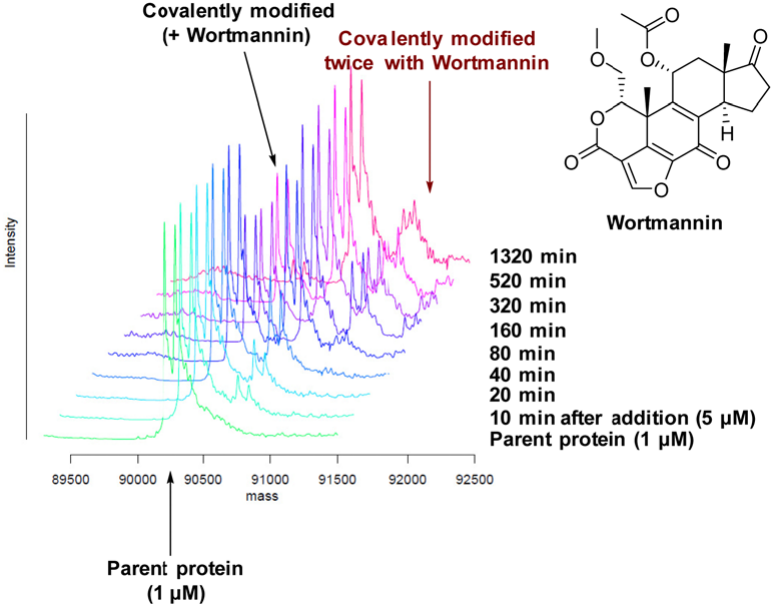
Time course of Wortmannin reacting with recombinant PI4KIIIβ.

There are 39 lysine residues in recombinant PI4KIIIβ which could lead to non-specific labelling of the protein with multiple warheads reacting. The incubation of an excess of 4 with the enzyme revealed only one covalent modification, indicative of a selective target engagement. Under the same mass spectrometry conditions, Wortmannin (5 μM) was profiled against PI4KIIIβ (1 μM) and the mass spectrum obtained is shown in Fig. 5. This showed multiple covalent adducts, highlighting the promiscuous covalent nature of Wortmannin in comparison to 4.^44^

**Fig. 6:**
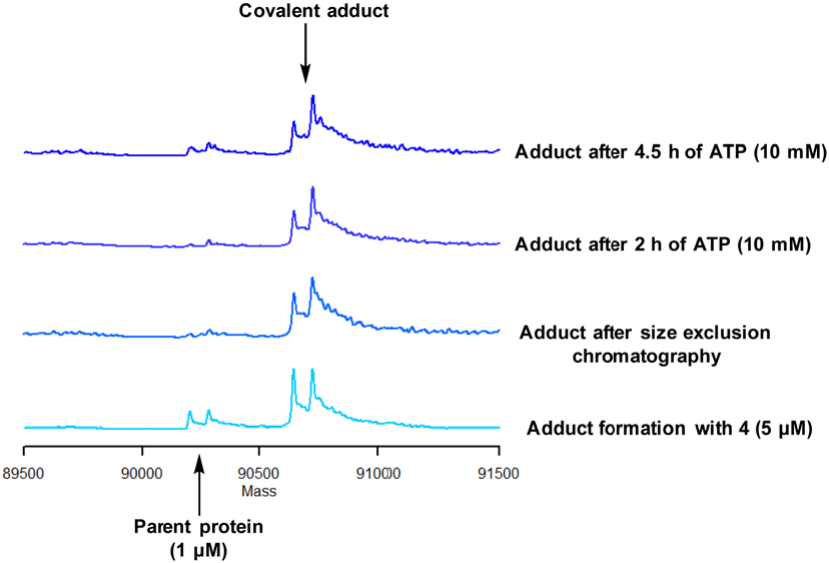
Stability of the covalent adduct between 4 (5 μM) and PI4KIIIβ in the presence of ATP (10 mM).

Protein mass spectrometry was also used to assess the stability of the covalent adduct between PI4KIIIβ and 4. Compound 4 (5 μM) was incubated with PI4KIIIβ (1 μM) and the adduct purified by size exclusion chromatography. The protein sample was then saturated with high concentrations of ATP (10 mM) and the solution monitored by mass spectrometry. Due to the high concentration of ATP, if the covalent step was reversible, ATP would compete in the active site of PI4KIIIβ reducing the extent of covalent modification. The data shown in Fig. 6 suggests that the adduct formed between 4 and PI4KIIIβ was stable under the conditions examined and not susceptible to competitive inhibition.

**Fig. 7:**
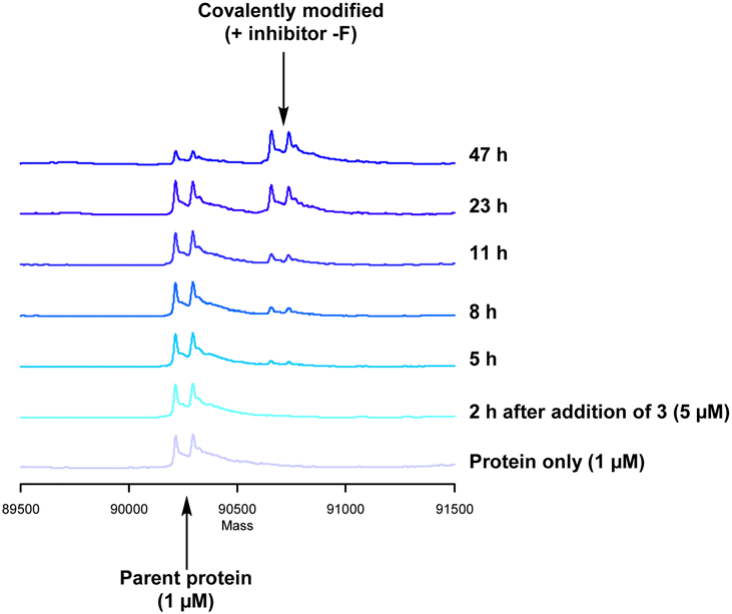
Time course for the reaction of 3 with recombinant PI4KIII.

Formation of the covalent adduct between 3 and PI4KIIIβ was profiled by mass spectrometry (Fig. 7). Interestingly, 3 displayed a significantly slower rate of covalent modification when compared to 4, reaching ∼50% conversion in 23 h. This suggests that the presence of an *o*-chloro substituent hindered the ability of this ligand to covalently link with PI4KIIIβ. Electronically, the presence of the *o*-chloro substituent would be expected to increase the electrophilicity of the sulfur centre, promoting reaction with the lysine residue. Therefore, the reduced rate of covalent modification was possibly due to an alternative conformational arrangement of 3, disfavouring nucleophilic attack. This is supported by the decreased potency of 3 when compared to 4 in the biological assays.

**Fig. 8:**
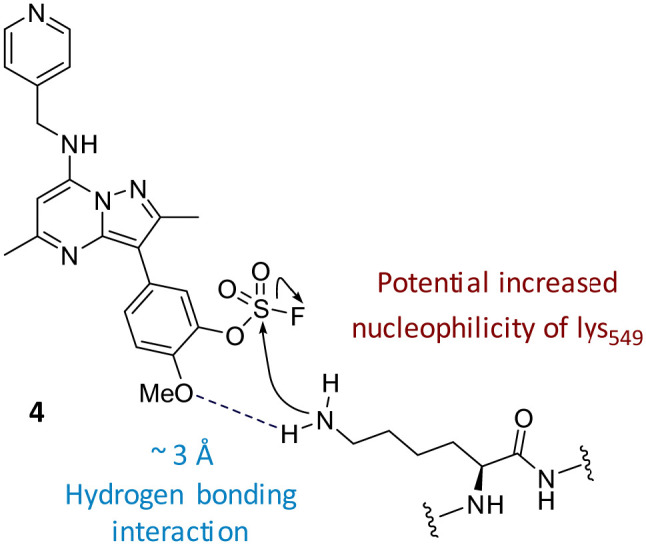
The *o*-methoxy group may increase the rate of covalent modification.

The data shown in Fig. 7 is in contrast to the expected relative electrophilicity of the fluorosulfate groups in compounds 3 and 4. The electron donating capability of the methoxy group in 4 decreases the electrophilicity of the fluorosulfate, reducing the expected rate of covalent modification. However, within the active site of PI4KIIIβ, the methoxy group increased the rate of covalent modification when compared to 3 (compare Fig. 5 and 7). It is possible that the methoxy group in 4 perturbs the p*K*_a_ of Lys_549_ augmenting its nucleophilicity and, as a result, increasing the rate of covalent modification (Fig. 8).

The rate of covalent modification of PI4KIIIβ was surprisingly slow, and this phenomenon is supported by recent literature around arylfluorosulfate compounds targeting lysine residues. For example, Pellecchia observed a slow onset of arylfluorosulfate containing inhibitors.^45^ These findings are consistent with a reduced reactivity of fluorosulfate containing inhibitors when compared to alternative electrophilic warheads.

Once a selective covalent adduct for PI4KIIIβ had been obtained, it was important to understand the different binding parameters that contributed to the potency of covalent inhibition. This was carried out using commercially available ADP-Glo assays.^46^ Achieving linearity of the high control (no compound) was difficult due to a prolonged onset of inhibition with 3. However, the measured onset of inhibition was reliably measured for compound 4 and the data is shown Table 2.

**Table 2:** Measured onset of inhibition of 4

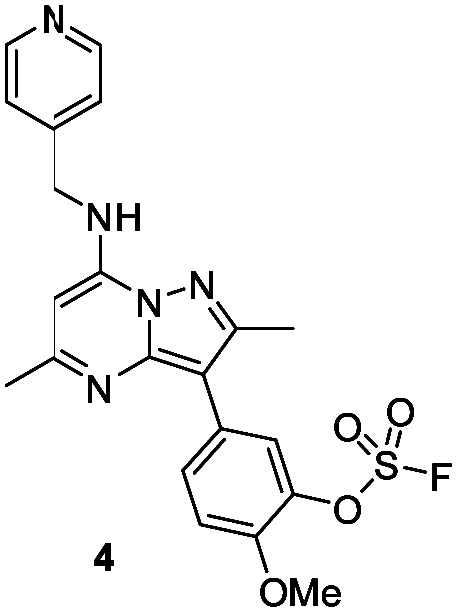		
*k*_I_ (M)	*K*_inact_ (s^−1^)	*K*_inact_/*K*_I_ (s^−1^M^−1^)
2.98 × 10^−8^	1.17 × 10^−4^	3.9 × 10^3^

The time course of the assay was significantly extended in comparison to previous literature methods.^26^ For smaller *K*_I_ values, the equilibrium is driven towards an initial protein inhibitor complex. The measured *K*_I_ was 29.8 nM which means the reversible interactions were favourable and a low concentration of 4 was required to achieve 50% inhibition. Despite this, the irreversible step, forming a covalent bond (described by *k*_inact_), was measured to be 1.17 × 10^−4^ s^−1^, which is orders of magnitude slower than literature precedent for a lipid kinase, thus, the rate of covalent bond formation was deemed to be slow.^26^ This observation could be due to the nucleophilicity of the lysine residue and/or the electrophilicity of the fluorosulfate. As a result, the overall observed rate described by *k*_inact_/*K*_I_ was slow. Compound 4 is therefore suitable for a slow-onset of inhibition of PI4KIIIβ with a prolonged duration of action.

**Fig. 9:**
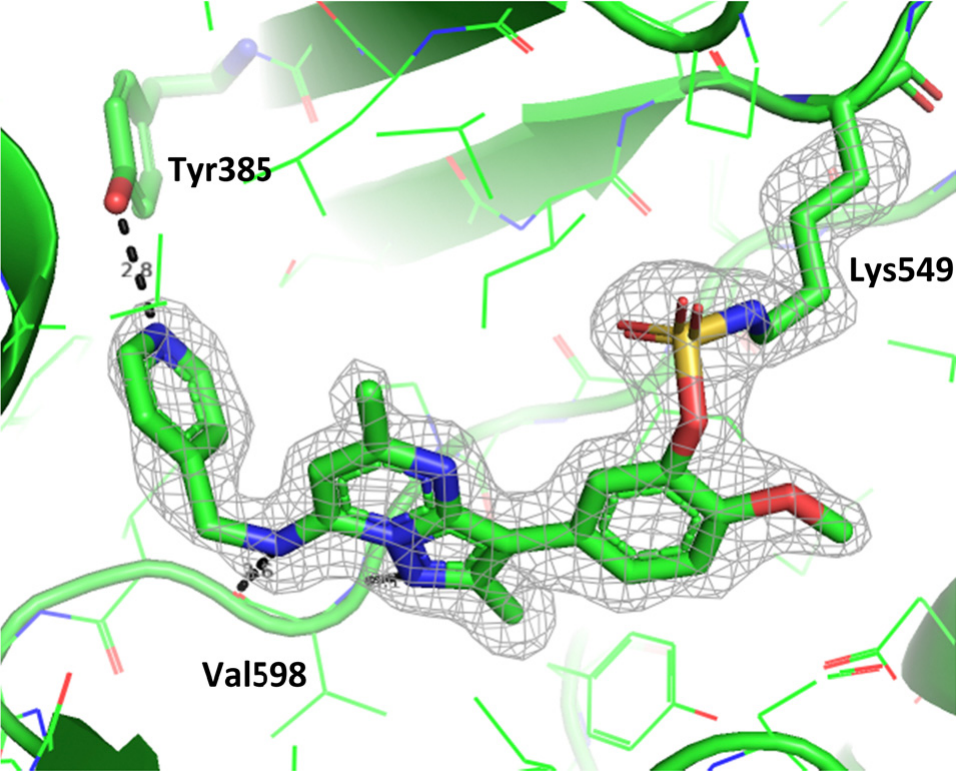
Crystal structure of PI4KIIIβ covalently modified on Lys_549_ with 4 and a corresponding *F*_o_ − *F*_c_ difference “omit” map contoured at 3σ (grey mesh) [PyMOL^47^ generated figure] (PDB: 8Q6F). The compound is present in the ATP binding site and makes key H-bonding interactions with Tyr_385_ and hinge residue Val_598_.

To gain further evidence that compound 4 covalently labelled Lys_549_, protein X-ray crystallography was used to characterise the adduct. Electron density consistent with 4 forming a covalent bond with Lys_549_ was observed (Fig. 9). The oxygen linker of the fluorosulfate group directed the warhead to interact with Lys_549_; it is possible that rotation about the fluorosulfate warhead was restricted due to a steric clash with the *o*-methoxy substituent. Compound 4 bound through a hinge binding interaction between the valine backbone amide and the nitrogen donor/acceptor atoms of the heterocyclic core. In addition, the *p*-pyridyl head group formed a favourable hydrogen bonding interaction with Tyr_385_.

The *p*-pyridyl head group of 4 formed a favourable hydrogen bonding interaction with Tyr_385_ (Fig. 9). Based on the reported reactivity of fluorosulfate warheads with tyrosine residues and the hydrogen bonding interaction displayed within the crystal structure, it was hypothesised that Tyr_385_ could be covalently modified with our PI4KIIIβ scaffold.^45,48,49^ To target this residue, 8 along with the control compounds 7 and 9 were prepared and evaluated for biological activity against the protein (Table 4).

**Fig. 10:**
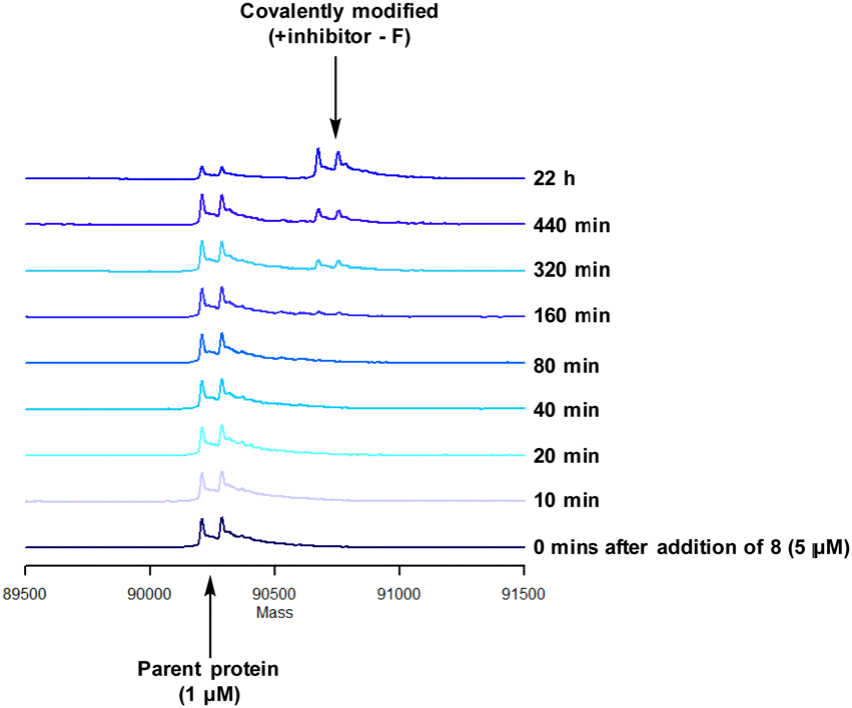
Reaction of 8 with recombinant PI4KIIIβ.

As shown in Table 3, 8 showed the same trend in activity as observed for 3 and 4, where a significant increase in potency was identified in progressing from the enzyme to the cell assay. Control compounds 7 and 9 retained their activity in the CPE assay rather than the value substantially increasing suggesting they were not covalent modifiers of the protein. It is important to note that potency in the cell assay for 7 was similar to the potency of 8. This may suggest that once 8 entered the cell it underwent hydrolysis to give phenol 7 which could be responsible for the observed increase. In order to probe this further, 8 was profiled by mass spectrometry (Fig. 10).

**Table 3:** Biological data for 8 and control compounds 7 and 9

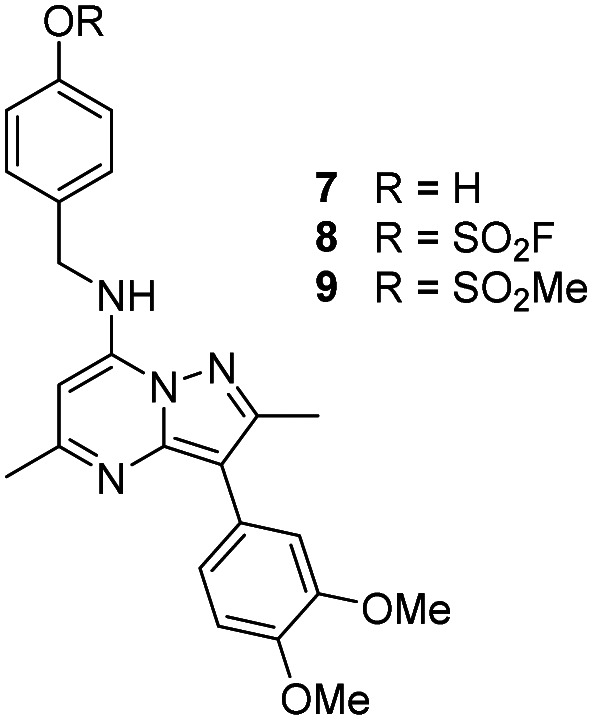			
	PI4KIIIβ pIC_50_^a^	CPE pIC_50_^b^	Δ(CPE-PI4KIIIβ)
7	7.4 ± 0.080	7.4 ± 0.105	0
8	6.3 ± 0.060	7.3 ± 0.085	1.0
9	6.5	6.7 ± 0.073	0.2

a Compound incubated with protein for 40 minutes.

b Compound incubated with cells for 48 hours.

A single covalent modification was detected between 8 and PI4KIIIβ with no evidence of multiple covalent adducts. It took approximately 22 h to reach ∼90% conversion showing a longer incubation time was required to engage with Tyr_385_ compared to that observed with 4 and Lys_549_, possibly due to the reduced nucleophilicity of the tyrosine residue. We therefore established the kinetics of the reversible and irreversible steps of the inhibition (Table 4).

**Table 4:** Measured onset of inhibition of 8

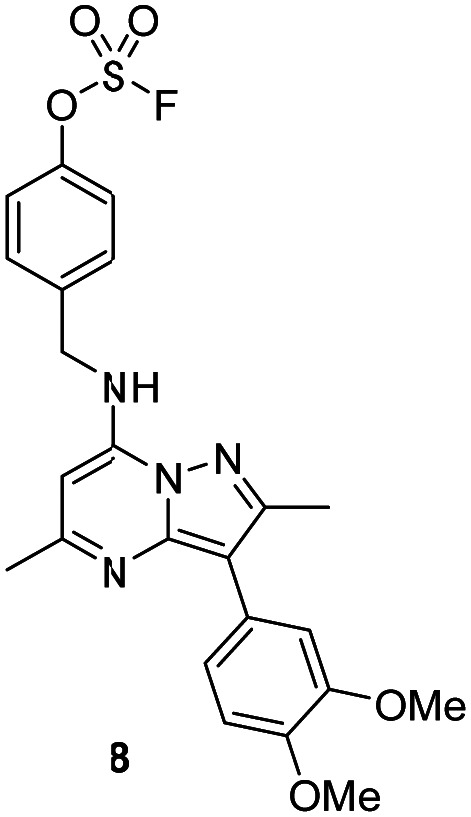		
*k*_I_ (M)	*K*_inact_ (s^−1^)	*K*_inact_/*K*_I_ (s^−1^ M^−1^)
1.35 × 10^−6^	≤1.6 × 10^−6^	≤1.2

Compound 8 was significantly slower in forming a covalent adduct with the protein in comparison to 4 as shown by mass spectrometry. The reversible binding for 8 was approximately ∼1.4 μM and the irreversible step was ≤1.6 × 10^−6^ s^−1^. This correlates to a higher concentration of compound being required to form a reversible complex in comparison to 4, showing the reversible interactions to be less favourable. In addition, covalent bond formation was two orders of magnitude slower for 8 in comparison to 4. As a result, the overall observed rate described by *k*_inact_/*K*_I_ for 8 was small (≤1.2) (Table 4).

**Fig. 11:**
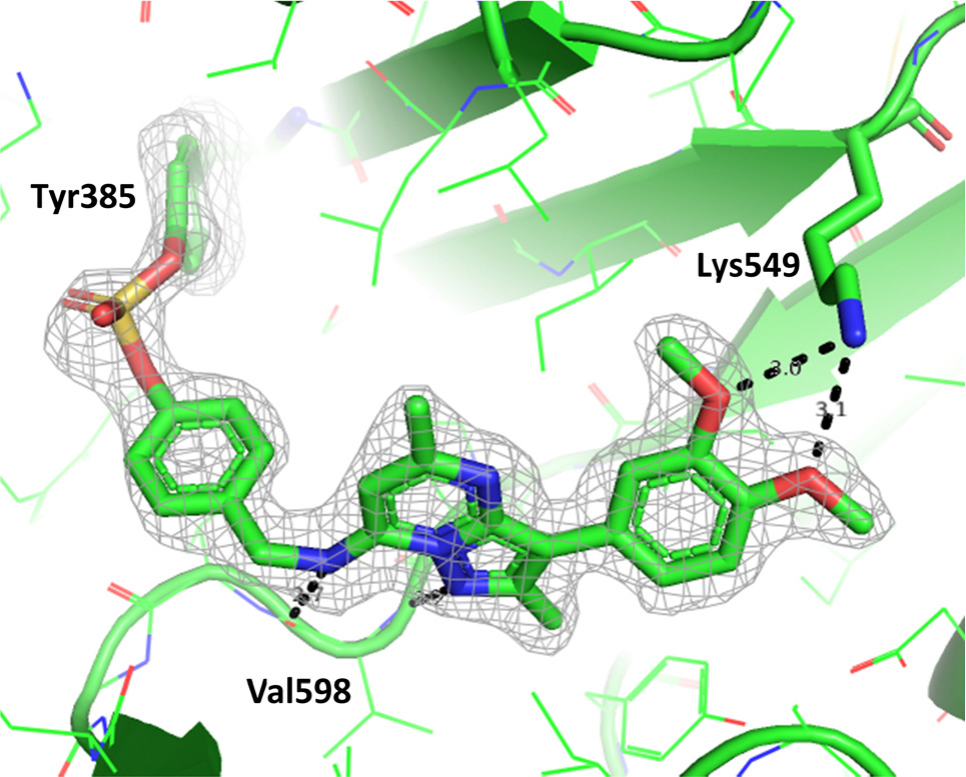
Crystal structure of PI4KIIIβ covalently modified on Tyr_385_ with 8 and a corresponding *F*_o_ − *F*_c_ difference “omit” map contoured at 3σ (grey mesh) [PyMOL^47^ generated figure] (PDB: 8Q6G). The compound is present in the ATP binding site and makes key H-bonding interactions with Lys_549_ and hinge residue Val_598_.

To determine the site of covalent modification, X-ray crystallography was carried out for 8, which showed Tyr_385_ was covalently modified (Fig. 11). A hinge binding interaction with the valine was observed as well as a bifurcated hydrogen bonding interaction with Lys_549_ through the two methoxy substituents. This highlights the versatility of the fluorosulfate group, where two different amino acids in the same active site were selectively covalently modified using this reactive warhead.

As 4 modified Lys_549_ and 8 modified Tyr_385_ we were intrigued to discover if a compound containing two fluorosulfate warheads could target both of these residues simultaneously; compound 11 was designed and synthesised alongside the bis-phenol control compound 10, which were profiled in the biochemical and CPE assays (Table 5).

**Table 5:** Biochemical data for 11 and control compound 10

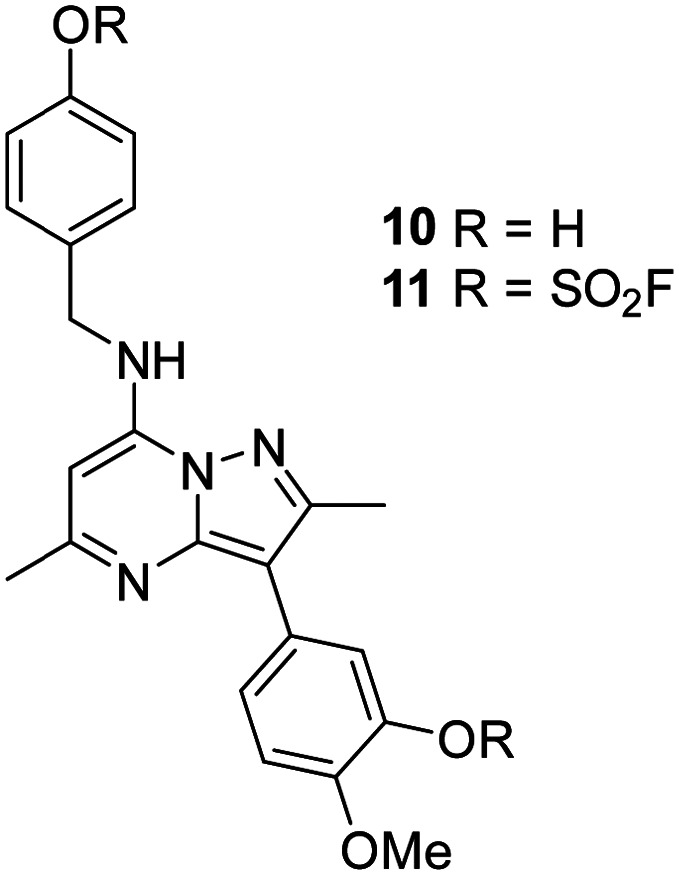			
	PI4KIIIβ pIC_50_^a^	CPE pIC_50_^b^	Δ(CPE-PI4KIIIβ)
10	6.6	6.4 ± 0.017	−0.2
11	<5.0 ± 0.00	6.0 ± 0.686^c^	>1.0

a Compound incubated with protein for 40 minutes.

b Compound incubated with cells for 48 hours.

c On one test occasion was inactive (<5).

**Fig. 12:**
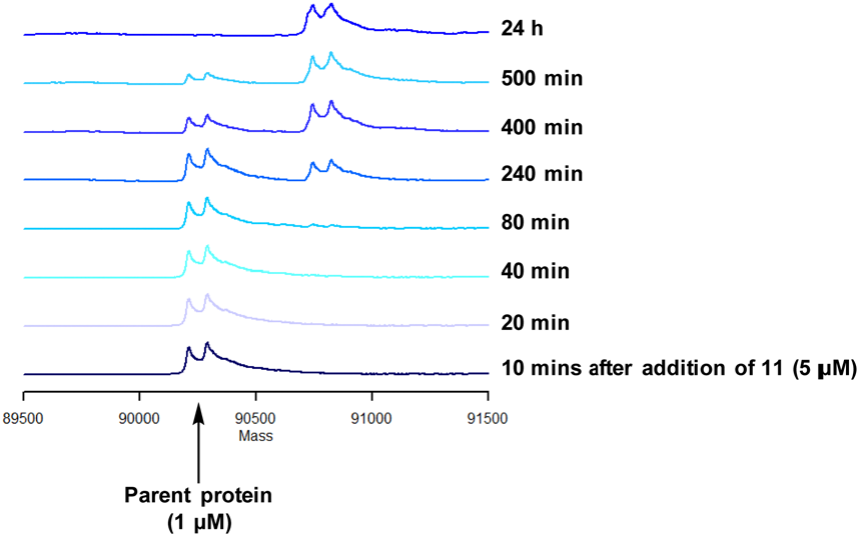
Reaction of 11 with recombinant PI4KIIIβ.

Compound 11 was inactive in the enzyme assay at the concentrations tested suggesting this bis-fluorosulfate may not be accommodated in the ATP binding pocket as effectively as 10. However, there was a marked potency increase observed in the cell assay, indicative of a time dependent inhibition of PI4KIIIβ for 11. To assess this possibility a mass spectrometry experiment was carried out which revealed a covalent adduct molecular ion (Fig. 12).

**Fig. 13:**
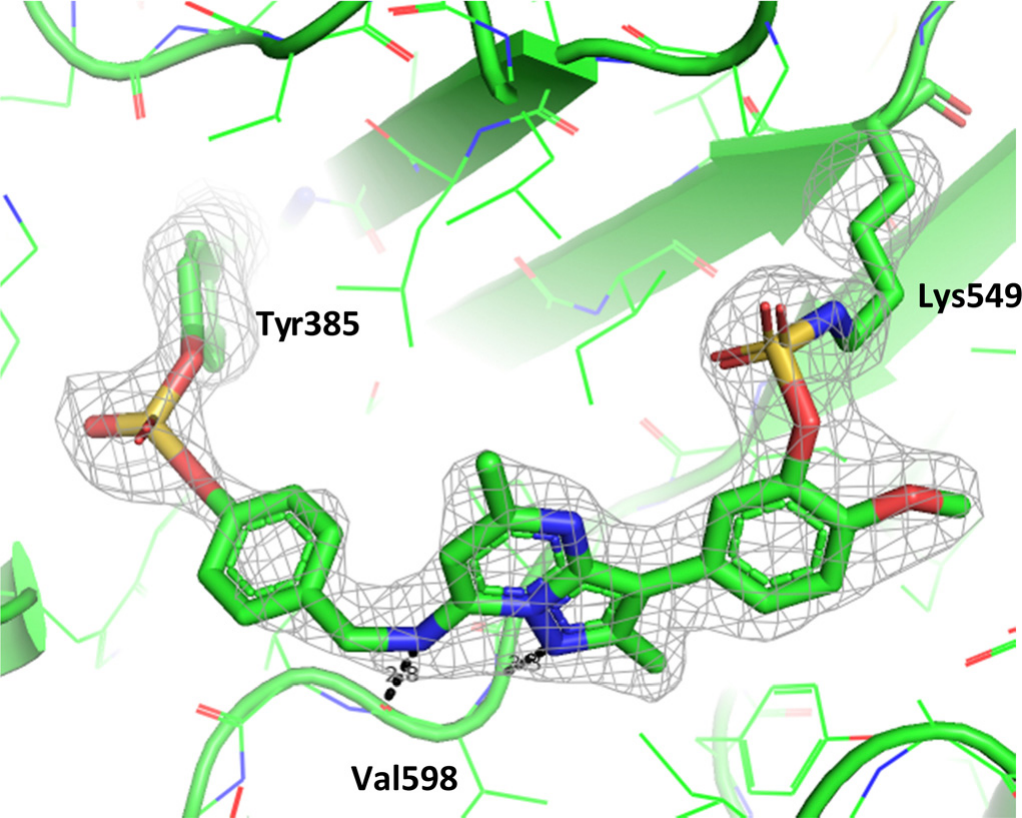
Crystal structure of 11 engaged with Lys_549_ and Tyr_385_ in PI4KIIIβ and a corresponding *F*_o_ − *F*_c_ difference “omit” map contoured at 3σ (grey mesh) [PyMOL^47^ generated figure] (PDB: 8Q6H). The compound is present in the ATP binding site and makes key H-bonding interactions with hinge residue Val_598_.

Compound 11 covalently modified PI4KIIIβ, fully labelling the protein after 24 h. The slow rate of covalent modification was expected due to the weaker binding affinity of the compound, suggesting a higher concentration of inhibitor would be necessary to form the initial reversible complex. Two covalent modifications were expected to take place and based on previous mass spectrometry and kinetic evidence, it was anticipated that Lys_549_ would react first, followed by Tyr_385_. To help investigate the possibility of a dual interaction with the protein, X-ray crystallography was used to characterise the adduct of 11 and PI4KIIIβ (Fig. 13).

Compound 11 adopted a dual covalent binding mode labelling both Lys_549_ and Tyr_385_ maintaining the direct H-bonding interactions at the hinge region of the protein. Adoption of a covalent strategy as a platform for drug discovery has been met with remarkable clinical success, specifically in the field of oncology.^50^ A specific challenge within the area is the development of drug resistance by mutation, leading to loss of efficacy.^17^ This can be avoided by targeting catalytic residues of the protein, which on mutation, result in loss of function.^50^ Targeting two sites within a protein of interest could provide an alternative approach to addressing this problem. To achieve success against these goals, the distance between the warheads, their trajectories relative to the nucleophilic protein residues along with the reactivities of the warhead would need to be optimised to ensure an appropriate compound profile. This would require substantial compound optimisation and presents a significant opportunity for future investigations. Overall, this work highlights the potential of the fluorosulfate group as a covalent modifier targeting non-cysteine residues in proteins, an emerging area of research in chemical biology and drug discovery.

### Chemistry

**Fig. 14:**
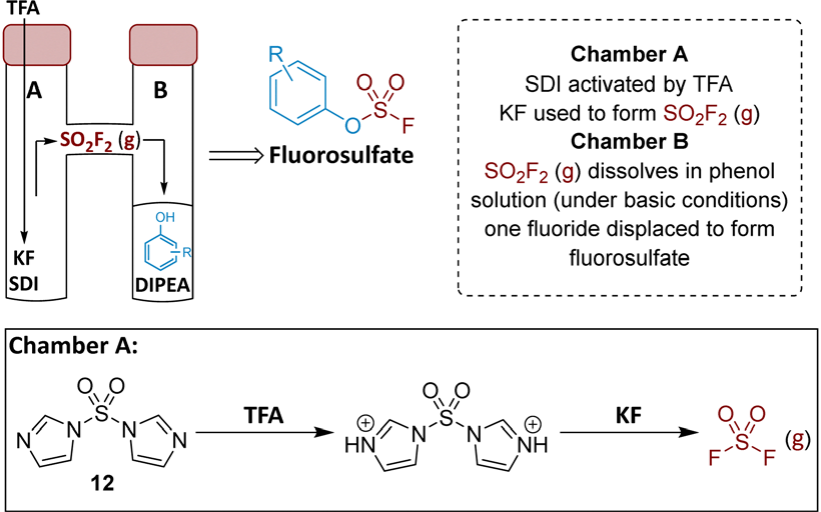
Use of COware to generate fluorosulfate compounds.

To prepare the fluorosulfate warheads, COware two-chamber glassware was used (Fig. 14).^36^ Sulfuryl fluoride (SO_2_F_2_) gas is highly toxic and corrosive, therefore, a sealed COware reactor system was used to construct the fluorosulfate warhead by generating the gas *in situ*. 1,1′-Sulfonyldiimidazole (SDI) 12, trifluoroacetic acid (TFA) and KF were added to chamber A generating SO_2_F_2(g)_ which transferred to chamber B and reacted with a phenol nucleophile under basic conditions to give the corresponding fluorosulfate.

**Scheme 1:**
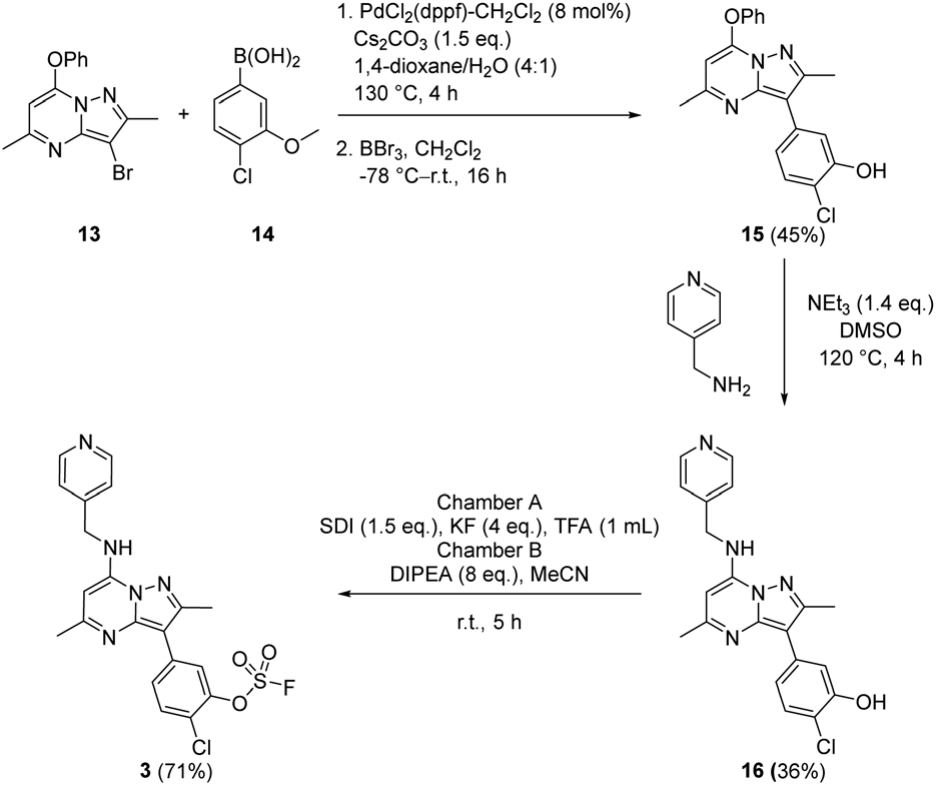
Synthesis of compound 3.

Suzuki coupling of the aryl bromide 13 and boronic acid 14 under conditions developed in previous work from our laboratories^41^ followed by treatment with BBr_3_ gave the intermediate 15 (45%) after purification by chromatography. S_N_Ar reaction with 4-(aminomethyl)pyridine gave the phenol 16 (36%) which was converted to the fluorosulfate 3 (71%) in a COware reactor (Scheme 1).

**Scheme 2:**
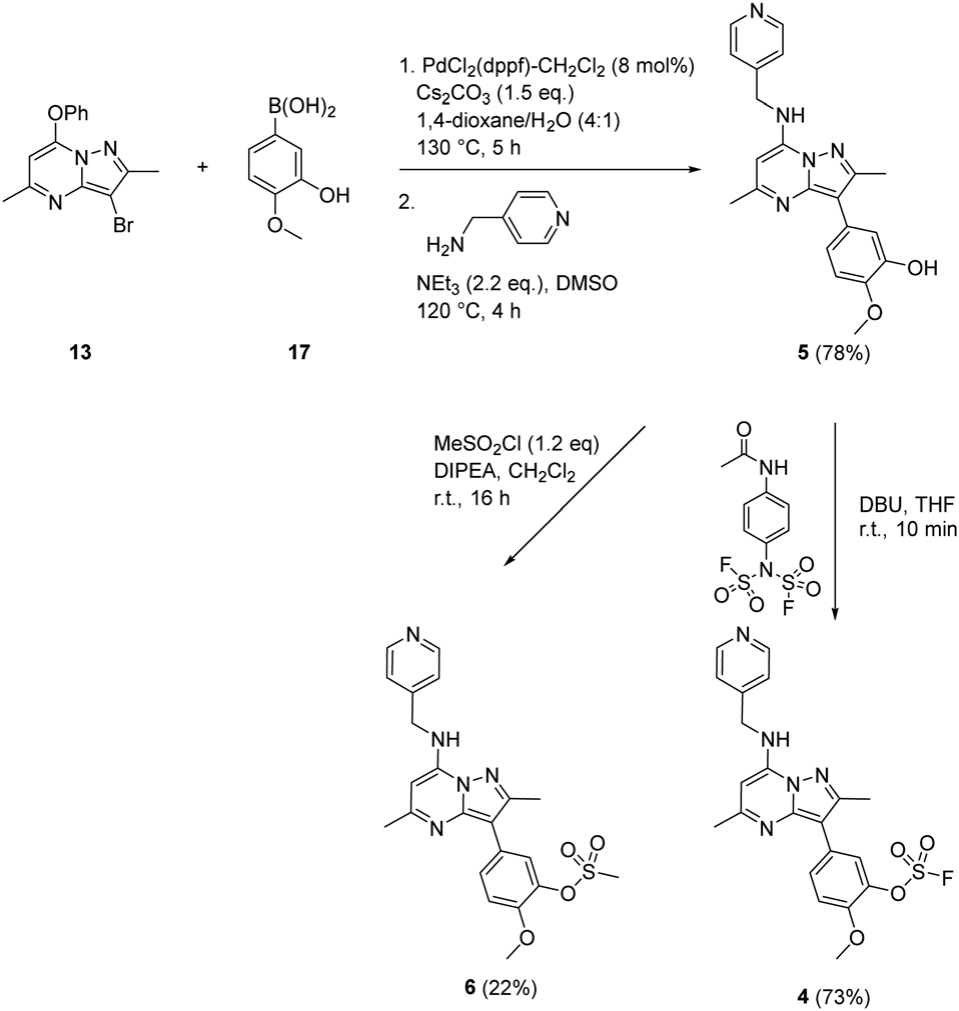
Synthesis of compounds 4, 5 and 6.

A similar strategy was adopted in the preparation of the target compounds 4, 5 and 6. Suzuki coupling followed by S_N_Ar reaction with 4-(aminomethyl)pyridine in the presence of triethylamine gave the phenol 5 (78%), which was converted into the corresponding mesylate 6 (22%) or the fluorosulfate 4 (73%)^51^ (Scheme 2).

**Scheme 3:**
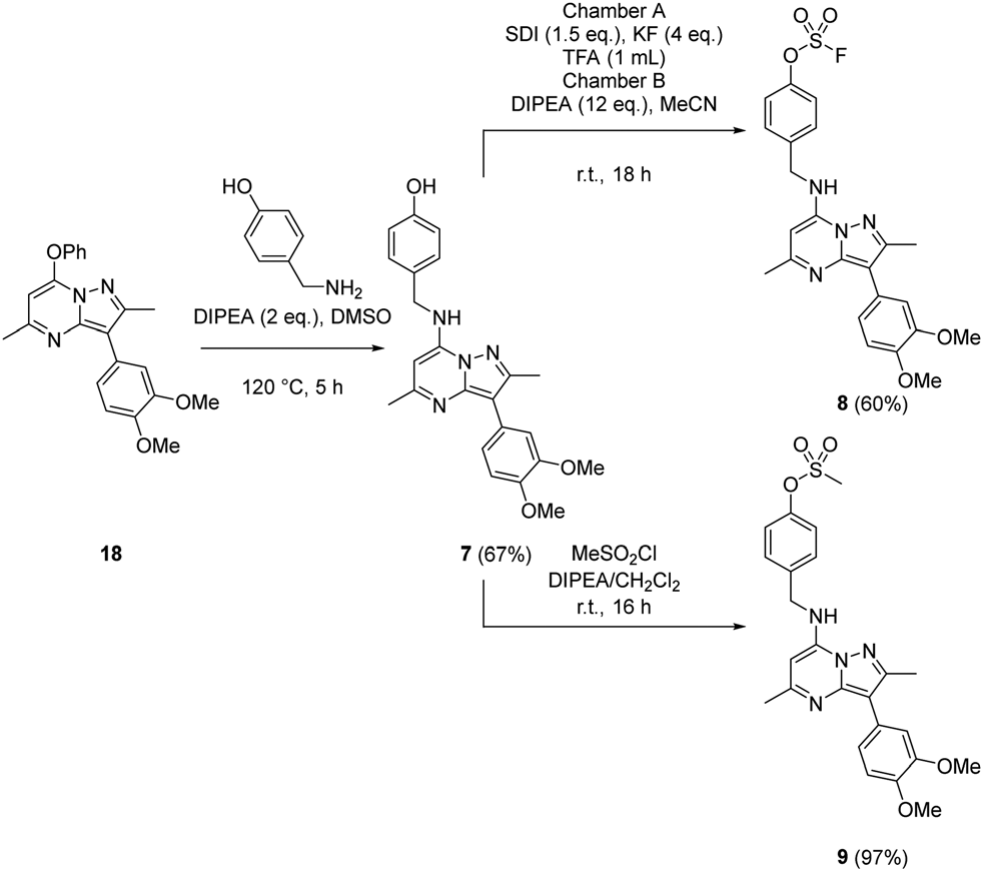
Synthesis of compounds 7, 8 and 9.

Probes to target Tyr_385_ were prepared from the known precursor 18 (Scheme 3).^52^ Treatment of 18 with 4-hydroxybenzylamine at 120 °C in the presence of DIPEA gave the adduct 7 (67%), which was converted to fluorosulfate 8 (60%) in a COware reactor and the methanesulfonate ester 9 (97%) by reaction with methanesulfonyl chloride under basic conditions.

**Scheme 4:**
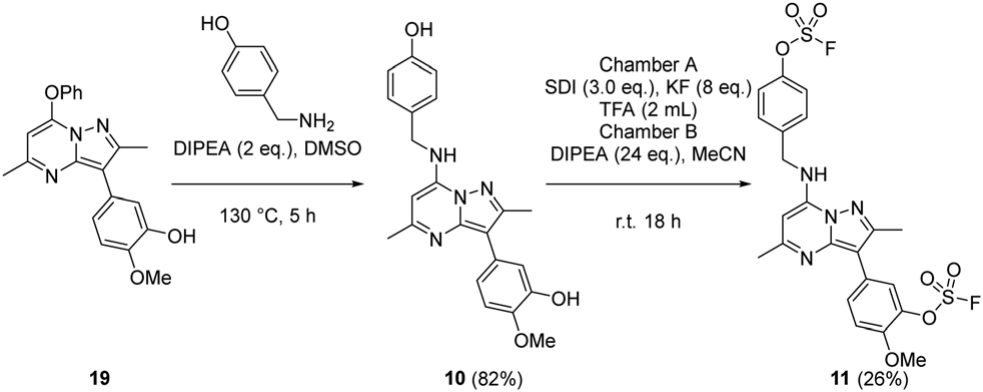
Synthesis of compounds 10 and 11.

The bivalent probe 11 and its phenolic precursor 10 were prepared using the same strategy starting with phenol 19*via* an S_N_Ar reaction followed by formation of the bisfluorosulfate 11 (26%) (Scheme 4).

## Conclusions

The aim of this project was to develop a selective irreversible covalent inhibitor of PI4KIIIβ which targeted the conserved lysine (Lys_549_) in the ATP binding site. Compounds with fluorosulfate warheads were designed and synthesised based upon a known reversible inhibitor of the protein. Compounds 3 and 4 displayed slow onset of inhibition, placing emphasis on initial reversible interactions with the receptor over the covalent modification resulting in an inherent selectivity for the protein. Further studies could use an alkynylated derivative of 4 to establish protein selectivity in a cellular environment.

This research confirms the hypothesis whereby the conserved lysine in the lipid kinome can be targeted through a covalent warhead attached to a selective scaffold. The crystal structure of 4 confirmed this and also indicated the potential for targeting covalent modification of a tyrosine residue (Tyr_385_) in the active site. Compound 8 was synthesised and a crystal structure showed that this residue could also be covalently modified. Building upon this exciting finding, the covalent warheads were combined to form the first dual covalent inhibitor 11 targeting both lysine (Lys_549_) and tyrosine (Tyr_385_) with X-ray crystallography supporting these proposed interactions. A dual covalent inhibitor targeting two cysteine residues has recently been described for FGFR4.^53^ With further optimisation and tailored reactivity of the warhead this strategy provides the potential to develop tools and drug molecules to overcome resistance mutations such as those frequently observed in the infectious disease and oncology areas. Targeting two non-cysteine residues significantly widens the scope of this approach to other proteins of interest.

## Experimental

### 3-(4-Chloro-3-methoxyphenyl)-2,5-dimethyl-7-phenoxypyrazolo[1,5-*a*]pyrimidine

A microwave vial (20 mL) was charged with 3-bromo-2,5-dimethyl-7-phenoxypyrazolo[1,5-*a*]pyrimidine (0.92 g, 2.89 mmol) (13), (4-chloro-3-methoxyphenyl)boronic acid (0.81 g, 4.34 mmol) (14), PdCl_2_(dppf)-CH_2_Cl_2_ adduct (0.189 g, 0.231 mmol), caesium carbonate (1.413 g, 4.34 mmol) and 1,4-dioxane (10 mL)/water (2.5 mL). The reaction vessel was sealed and heated in a Biotage Initiator microwave at 130 °C for 4 h. After cooling the reaction, the reaction mixture was passed through Celite™, diluted with ethyl acetate and washed with brine. The organic layer was passed through a hydrophobic frit and concentrated *in vacuo* to give the title compound 3-(4-chloro-3-methoxyphenyl)-2,5-dimethyl-7-phenoxypyrazolo[1,5-*a*]pyrimidine (1.054 g, 2.77 mmol, 96%) which was used in the next experiment without further purification. ^1^H NMR (400 MHz, DMSO-d_6_) *δ* = 7.62–7.42 (m, 7H), 7.38 (d, *J* = 2.0 Hz, 1H), 6.00 (s, 1H), 3.93 (s, 3H), 2.61 (s, 3H), 2.44 (s, 3H). ^13^C NMR (101 MHz, DMSO-d_6_) *δ* = 161.6, 154.8, 154.2, 152.4, 152.2, 147.9, 133.2, 131.2, 130.1, 127.4, 121.5, 121.3, 118.9, 113.0, 106.4, 91.1, 56.4, 25.3, 15.1. LCMS (formic) 97%; *t*_ret_ = 1.19 min, [M + H]^+^ = 376.3.

### 2-Chloro-5-(2,5-dimethyl-7-phenoxypyrazolo[1,5-*a*]pyrimidin-3-yl)phenol (15)

A flask was charged with 3-(4-chloro-3-methoxyphenyl)-2,5-dimethyl-7-phenoxypyrazolo[1,5-*a*]pyrimidine (100 mg, 0.26 mmol) and anhydrous CH_2_Cl_2_ (6 mL). The solution was stirred in an acetone-dry ice bath, before adding boron tribromide solution in CH_2_Cl_2_ (0.360 mL, 2.11 mmol) and the reaction was left to stir at r.t. for 16 h. The reaction mixture was diluted with CH_2_Cl_2_ and washed with saturated aqueous sodium bicarbonate solution. The organic layer was concentrated *in vacuo* and purified by MDAP (Method D). The appropriate fractions were combined and evaporated *in vacuo* to give 2-chloro-5-(2,5-dimethyl-7-phenoxypyrazolo[1,5-*a*]pyrimidin-3-yl)phenol 15 (43 mg, 0.12 mmol, 45% yield). ^1^H NMR (400 MHz, DMSO-d_6_) *δ* = 7.58 (m, 2H), 7.47–7.42 (m, 4H), 7.39 (d, *J* = 8.3 Hz, 1H), 7.23–7.18 (m, 1H), 5.97 (s, 1H), 2.55 (s, 3H), 2.43 (s, 3H) (OH signal not observed). ^13^C NMR (101 MHz, DMSO-d_6_) *δ* = 161.3, 154.2, 153.6, 152.3, 152.2, 147.8, 132.7, 131.2, 130.0, 127.4, 121.4, 120.3, 117.8, 117.0, 106.6, 90.9, 25.2, 15.0. LCMS (HpH) 100%; *t*_ret_ = 1.20 min, [M + H]^+^ 366.1. HRMS (C_20_H_16_ClN_3_O_2_) [M + H]^+^ requires 366.1009 found [M + H]^+^ 366.1004.

### 2-Chloro-5-(2,5-dimethyl-7-((pyridin-4-ylmethyl)amino)pyrazolo[1,5-*a*]pyrimidin-3-yl)phenol (16)

A microwave vial (5 mL) was charged with 2-chloro-5-(2,5-dimethyl-7-phenoxypyrazolo[1,5-*a*]pyrimidin-3-yl)phenol 15 (43 mg, 0.12 mmol) (15), pyridin-4-ylmethanamine (0.013 mL, 0.13 mmol), triethylamine (0.025 mL, 0.18 mmol) and DMSO (3 mL). The reaction vessel was sealed and heated in a microwave at 120 °C for 4 h. After cooling, the reaction mixture was diluted with EtOAc and washed with saturated aqueous sodium bicarbonate solution. The organic layer was concentrated *in vacuo* and purified by MDAP (Method E). The appropriate fractions were combined and concentrated *in vacuo* to give 2-chloro-5-(2,5-dimethyl-7-((pyridin-4-ylmethyl)amino)pyrazolo [1,5-*a*]pyrimidin-3-yl)phenol 16 (16 mg, 0.04 mmol, 36% yield). ^1^H NMR (400 MHz, DMSO-d_6_) *δ* = 8.55–8.47 (m, 3H), 7.44 (d, *J* = 2.0 Hz, 1H), 7.40–7.36 (m, 2H), 7.34 (d, *J* = 8.3 Hz, 1H), 7.21 (dd, *J* = 2.0, 8.3 Hz, 1H), 5.99 (s, 1H), 4.65 (d, *J* = 6.5 Hz, 2H), 2.55 (s, 3H), 2.34 (s, 3H) (OH signal not observed). ^13^C NMR (101 MHz, DMSO-d_6_) *δ* = 159.5, 153.3, 150.6, 150.2, 147.8, 146.5, 146.3, 133.6, 129.8, 122.4, 120.3, 117.1, 116.7, 105.1, 86.3, 43.9, 25.3, 15.1. LCMS (formic) 97%; *t*_ret_ = 0.52 min, [M + H]^+^ 380.4. HRMS (C_20_H_18_ClN_5_O) [M + H]^+^ requires 380.1278 found [M + H]^+^ 380.1282.

### 2-Chloro-5-(2,5-dimethyl-7-((pyridin-4-ylmethyl)amino)pyrazolo[1,5-*a*]pyrimidin-3-yl)phenyl sulfofluoridate (3)

Chamber A of the COware flask reactor was charged with sulfonyldiimidazole (258 mg, 1.30 mmol) (12) and potassium fluoride (202 mg, 3.48 mmol). Chamber B of the COware flask reactor was charged with 2-chloro-5-(2,5-dimethyl-7-((pyridin-4-ylmethyl)amino)pyrazolo[1,5-*a*]pyrimidin-3-yl)phenol (330 mg, 0.87 mmol) (16), DIPEA (1.21 mL, 6.95 mmol) and acetonitrile (4 mL). Trifluoroacetic acid (1 mL) was added to chamber A *via* syringe injection. The reaction mixture was stirred vigorously for 5 h at room temperature, chamber B was decanted and chamber A was quenched with concentrated aqueous NaOH. The reaction mixture was concentrated *in vacuo* and purified by MDAP (Method A). The appropriate fractions were combined and concentrated *in vacuo* to afford 2-chloro-5-(2,5-dimethyl-7-((pyridin-4-ylmethyl)amino) pyrazolo[1,5-*a*]pyrimidin-3-yl)phenyl sulfofluoridate as a colourless oil 3 (282 mg, 0.62 mmol, 71% yield). ^1^H NMR (400 MHz, DMSO-d_6_) *δ* = 9.05–8.97 (m, 1H), 8.80 (d, *J* = 6.5 Hz, 2H), 8.29–8.26 (m, 1H), 7.97–7.82 (m, 4H), 6.22 (s, 1H), 4.93 (d, *J* = 6.5 Hz, 2H), 2.65–2.61 (m, 3H), 2.40–2.37 (m, 3H). ^13^C NMR (DMSO-d_6_, 151 MHz) *δ* = 160.0, 151.7, 146.8, 145.5, 144.3, 134.9, 131.7, 129.7, 124.6, 121.9, 117.2, 115.3, 102.9, 87.4, 44.4, 24.6, 15.0 (one carbon signal not observed). ^19^F NMR (376 MHz, DMSO-d_6_) *δ* = 41.57 (s, 1F). LCMS (formic) 100%; *t*_ret_ = 0.92 min, [M + H]^+^ 462.3. HRMS (C_20_H_17_ClFN_5_O_3_S) [M + H]^+^ requires 462.0803 found [M + H]^+^ 462.0803.

### 5-(2,5-Dimethyl-7-phenoxypyrazolo[1,5-*a*]pyrimidin-3-yl)-2-methoxyphenol

The microwave vial (20 mL) was charged with 3-bromo-2,5-dimethyl-7-phenoxypyrazolo[1,5-*a*]pyrimidine (260 mg, 0.82 mmol) (13), 2-methoxy-5-(4,4,5,5-tetramethyl-1,3,2-dioxaborolan-2-yl)phenol (225 mg, 0.90 mmol) (17), PdCl_2_(dppf)-CH_2_Cl_2_adduct (53.4 mg, 0.07 mmol), caesium carbonate (399 mg, 1.23 mmol) in 1,4-dioxane (8 mL) and water (2 mL). The reaction vessel was sealed and heated in a microwave at 130 °C for 5 h. After cooling, the reaction mixture was passed through Celite™ and concentrated *in vacuo*. The crude product was purified by column chromatography eluting with EtOAc in cyclohexane (0–60% gradient). The appropriate fractions were combined and concentrated *in vacuo* to give 5-(2,5-dimethyl-7-phenoxypyrazolo[1,5-*a*]pyrimidin-3-yl)-2-methoxyphenol (290 mg, 0.80 mmol, 98% yield). ^1^H NMR (400 MHz, DMSO-d_6_) *δ* = 8.98 (br s, 1H), 7.58 (t, *J* = 7.1 Hz, 2H), 7.46–7.40 (m, 3H), 7.24–7.22 (m, 1H), 7.13–7.08 (m, 1H), 7.03–6.99 (m, 1H), 5.91 (s, 1H), 3.82 (s, 3H), 2.54 (s, 3H), 2.41 (s, 3H). ^13^C NMR (DMSO-d_6_, 101 MHz) *δ* = 160.6, 154.1, 152.3, 152.1, 147.6, 146.8, 146.6, 131.1, 129.8, 127.3, 125.6, 121.3, 120.0, 116.7, 115.7, 112.9, 90.5, 56.2, 55.3, 25.2, 14.8. LCMS (formic) 90%; *t*_ret_ = 1.05 min, [M + H]^+^ 362.3. HRMS (C_21_H_19_N_3_O_3_) [M + H]^+^ requires 362.1426 found [M + H]^+^ 362.1507.

### 5-(2,5-Dimethyl-7-((pyridin-4-ylmethyl)amino)pyrazolo[1,5-*a*]pyrimidin-3-yl)-2-methoxyphenol (5)

A microwave vial (20 mL) was charged with 5-(2,5-dimethyl-7-phenoxypyrazolo[1,5-*a*]pyrimidin-3-yl)-2-methoxyphenol (1.04 g, 2.88 mmol), pyridin-4-ylmethanamine (0.321 mL, 3.17 mmol), triethylamine (0.88 mL, 6.33 mmol) and DMSO (10 mL). The reaction vessel was sealed and heated in a microwave at 120 °C for 4 h. After cooling, the reaction mixture was diluted with EtOAc (20 mL) and washed with saturated aqueous sodium bicarbonate solution (20 mL). The organic layer was concentrated *in vacuo* and purified by column chromatography eluting with EtOAc in cyclohexane (0–100% gradient). The appropriate fractions were combined and concentrated *in vacuo* to give 5-(2,5-dimethyl-7-((pyridin-4-ylmethyl)amino)pyrazolo[1,5-*a*]pyrimidin-3-yl)-2-methoxyphenol 5 (865 mg, 2.31 mmol, 80% yield) as a colourless oil. ^1^H NMR (400 MHz, DMSO-d_6_) *δ* = 8.55–8.50 (m, 2H), 8.47–8.42 (m, 1H), 8.18 (s, 1H), 7.37 (d, *J* = 5.9 Hz, 2H), 7.20 (d, *J* = 2.4 Hz, 1H), 7.12–7.05 (m, 1H), 6.97 (d, *J* = 8.3 Hz, 1H), 5.93 (s, 1H), 4.64 (d, *J* = 6.4 Hz, 2H), 3.80 (s, 3H), 2.52 (s, 3H), 2.31 (s, 3H) (OH signal not observed). ^13^C NMR (DMSO-d_6_, 101 MHz) *δ* = 160.6, 154.1, 152.1, 150.2, 147.6, 146.8, 146.7, 131.1, 127.4, 125.6, 121.3, 116.6, 112.9, 94.9, 90.5, 89.9, 56.2, 25.2, 14.8. LCMS (formic) 97%; *t*_ret_ = 0.87 min, [M + H]^+^ 376.3. HRMS (C_21_H_21_N_5_O_2_) [M + H]^+^ requires 376.1773 found [M + H]^+^ 376.1769.

### 5-(2,5-Dimethyl-7-((pyridin-4-ylmethyl)amino)pyrazolo[1,5-*a*]pyrimidin-3-yl)-2-methoxyphenyl sulfofluoridate (4)

A microwave vial (5 mL) was charged with 5-(2,5-dimethyl-7-((pyridin-4-ylmethyl)amino)pyrazolo[1,5-*a*]pyrimidin-3-yl)-2-methoxyphenol (203 mg, 0.54 mmol) (5) and THF (3 mL). To this, (4-acetamidophenyl)(fluorosulfonyl)sulfamoyl fluoride (204 mg, 0.65 mmol) was added, followed by the addition of (*Z*)-3,4,5,6,8,9,10,11-octahydro-2*H*-pyrido[1,2-*a*][1,3]diazocine (0.2 mL, 1.19 mmol). The reaction was stirred for 10 minutes at room temperature. The reaction mixture was concentrated *in vacuo* and the residue was dissolved in CH_2_Cl_2_ (20 mL) and washed with brine (10 mL). The organic layer was concentrated *in vacuo* and the crude product was purified by MDAP (Method A). The appropriate fractions were combined and concentrated *in vacuo* to give 5-(2,5-dimethyl-7-((pyridin-4-ylmethyl)amino)pyrazolo[1,5-*a*]pyrimidin-3-yl)-2-methoxyphenyl sulfofluoridate 4 (179 mg, 0.40 mmol, 73% yield). ^1^H NMR (400 MHz, DMSO-d_6_) *δ* = 8.74 (br s, 1H), 8.64–8.57 (m, 2H), 7.96 (d, *J* = 1.8 Hz, 1H), 7.82 (dd, *J* = 2.1, 8.7 Hz, 1H), 7.51 (d, *J* = 6.3 Hz, 2H), 7.42 (d, *J* = 8.8 Hz, 1H), 6.07 (s, 1H), 4.73 (d, *J* = 6.5 Hz, 2H), 3.98–3.91 (m, 3H), 2.57 (s, 3H), 2.34 (s, 3H). ^13^C NMR (101 MHz, DMSO-d_6_) *δ* = 159.5, 151.0, 148.6, 148.4, 146.6, 141.2, 138.6, 129.9, 126.8, 123.1, 121.8, 114.8, 103.8, 86.6, 76.9, 57.0, 44.1, 24.9, 14.7. ^19^F NMR (376 MHz, DMSO-d_6_) *δ* = 40.35 (s, 1F). LCMS (formic); *t*_ret_ = 0.74 min, [M + H]^+^ 458.1.

### 5-(2,5-Dimethyl-7-((pyridin-4-ylmethyl)amino)pyrazolo[1,5-*a*]pyrimidin-3-yl)-2-methoxyphenyl methanesulfonate (6)

A microwave vial (5 mL) was charged with 5-(2,5-dimethyl-7-((pyridin-4-ylmethyl)amino)pyrazolo[1,5-*a*]pyrimidin-3-yl)-2-methoxyphenol (50 mg, 0.13 mmol) (5), methanesulfonyl chloride (0.012 mL, 0.16 mmol), DIPEA (0.047 mL, 0.27 mmol) and CH_2_Cl_2_ (2 mL). The reaction was stirred at r.t. for 16 h, diluted with CH_2_Cl_2_ and washed with brine. The organic layer was concentrated *in vacuo* and purified by column chromatography eluting with EtOAc/ethanol (3 : 1). The appropriate fractions were combined and concentrated *in vacuo* to give 5-(2,5-dimethyl-7-((pyridin-4-ylmethyl)amino)pyrazolo[1,5-*a*]pyrimidin-3-yl)-2-methoxyphenyl methanesulfonate 6 (13 mg, 0.03 mmol, 22% yield). ^1^H NMR (400 MHz, DMSO-d_6_) *δ* = 8.56–8.51 (m, 3H), 7.72–7.67 (m, 2H), 7.39–7.35 (m, 2H), 7.31–7.27 (m, 1H), 5.99 (s, 1H), 4.66 (d, *J* = 6.9 Hz, 2H), 3.89 (s, 3H), 3.39 (s, 3H), 2.57 (s, 3H), 2.33 (s, 3H). ^13^C NMR (101 MHz, DMSO-d_6_) *δ* = 159.5, 150.0, 149.6, 148.0, 146.4, 138.2, 128.1, 123.6, 122.5, 114.1, 110.0, 104.5, 86.3, 56.5, 43.9, 38.7, 25.2, 14.8. LCMS (formic) *t*_ret_ = 0.52 min, [M + H]^+^ 454.4. HRMS (C_22_H_23_N_5_O_4_S) [M + H]^+^ requires 454.1549 found [M + H]^+^ 454.1551.

### 4-(((3-(3,4-Dimethoxyphenyl)-2,5-dimethylpyrazolo[1,5-*a*]pyrimidin-7-yl)amino)methyl)phenol (7)

A microwave vial was charged with 3-(3,4-dimethoxyphenyl)-2,5-dimethyl-7-phenoxypyrazolo[1,5-*a*]pyrimidine (500 mg, 1.33 mmol) (18), 4-(aminomethyl)phenol (246 mg, 2.0 mmol) and DIPEA (0.465 mL, 2.66 mmol). The reaction vessel was sealed and heated in a microwave at 120 °C for 5 h. After cooling, the reaction mixture was diluted with EtOAc and washed with brine. The organic layer was concentrated *in vacuo* and purified by column chromatography eluting with EtOAc gradient (0–50%) cyclohexane. The appropriate fractions were combined and concentrated *in vacuo* to give 4-(((3-(3,4-dimethoxyphenyl)-2,5-dimethylpyrazolo[1,5-*a*]pyrimidin-7-yl)amino)methyl)phenol 7 (361 mg, 0.89 mmol, 67% yield). ^1^H NMR (400 MHz, DMSO-d_6_) *δ* = 9.31 (s, 1H), 8.22 (s, 1H), 7.42 (d, *J* = 2.0 Hz, 1H), 7.27–7.19 (m, 3H), 7.01 (d, *J* = 8.4 Hz, 1H), 6.75–6.71 (m, 2H), 5.99 (s, 1H), 4.47 (d, *J* = 6.4 Hz, 2H), 3.80 (s, 3H), 3.79 (s, 3H), 2.54 (s, 3H), 2.34 (s, 3H). ^13^C NMR (101 MHz, DMSO-d_6_) *δ* = 158.9, 157.0, 150.2, 149.0, 147.3, 146.3, 146.3, 128.9, 126.5, 120.9, 115.7, 113.1, 112.6, 105.9, 86.0, 56.1, 56.0, 44.5, 25.4, 14.9 (1 carbon signal not observed). LCMS (formic); *t*_ret_ = 1.08 min, [M + H]^+^ 405.3. HRMS (C_23_H_24_N_4_O_3_) [M + H]^+^ requires 405.1927 found [M + H]^+^ 405.1928.

### 4-(((3-(3,4-Dimethoxyphenyl)-2,5-dimethylpyrazolo[1,5-*a*]pyrimidin-7-yl)amino)methyl)phenyl sulfofluoridate (8)

Chamber A of the COware flask reactor was charged with sulfonyldiimidazole (110 mg, 0.55 mmol) (12) and potassium fluoride (86 mg, 1.47 mmol). Chamber B of the COware flask reactor was charged with 4-(((3-(3,4-dimethoxyphenyl)-2,5-dimethylpyrazolo [1,5-*a*]pyrimidin-7-yl)amino)methyl)phenol (149 mg, 0.37 mmol) (7), DIPEA (0.77 mL, 4.42 mmol) and MeCN (3 mL). Trifluoroacetic acid (1 mL) was added to chamber A *via* syringe injection and the reaction was stirred at room temperature for 18 h. After pressure release, chamber B was decanted and chamber A was quenched with aqueous concentrated NaOH. The reaction mixture was concentrated *in vacuo*, diluted with CH_2_Cl_2_ and washed with brine. The organic layer was concentrated *in vacuo* and purified by MDAP (Method B). The appropriate vials were combined and concentrated *in vacuo* to afford 4-(((3-(3,4-dimethoxyphenyl)-2,5-dimethylpyrazolo[1,5-*a*]pyrimidin-7-yl)amino)methyl)phenyl sulfofluoridate 8 (107 mg, 0.22 mmol, 60% yield). ^1^H NMR (600 MHz, DMSO-d_6_) *δ* = 8.55–8.43 (m, 1H), 7.63–7.55 (m, 4H), 7.40 (d, *J* = 1.8 Hz, 1H), 7.22 (dd, *J* = 1.8, 8.4 Hz, 1H), 7.02 (d, *J* = 8.4 Hz, 1H), 6.04 (s, 1H), 4.68 (d, *J* = 6.6 Hz, 2H), 3.80 (s, 3H), 3.79 (s, 3H), 2.55 (s, 3H), 2.34 (s, 3H). ^13^C NMR (151 MHz, DMSO-d_6_) *δ* = 163.4, 159.1, 150.4, 149.1, 149.0, 147.3, 146.3, 140.3, 129.8, 126.4, 121.6, 121.0, 113.1, 112.6, 106.0, 85.9, 56.1, 56.0, 44.0, 31.2, 14.9. ^19^F NMR (376 MHz, DMSO-d_6_) *δ* = 38.46 (s, 1F). LCMS (formic) 100%; *t*_ret_ = 0.94 min, [M + H]^+^ 487.1. HRMS (C_23_H_23_FN_4_O_5_S) [M + H]^+^ requires 487.1451 found [M + H]^+^ 487.1454. *ν*_max_ (neat)/cm^−1^ 3370, 2933, 1617, 1582, 1504, 1446, 1329, 1251, 1233, 1141, 1027, 915, 771.

### 4-(((3-(3,4-Dimethoxyphenyl)-2,5-dimethylpyrazolo[1,5-*a*]pyrimidin-7-yl)amino)methyl)phenyl methanesulfonate (9)

A microwave vial (5 mL) was charged with 4-(((3-(3,4-dimethoxyphenyl)-2,5-dimethylpyrazolo[1,5-*a*]pyrimidin-7-yl)amino)methyl)phenol (71 mg, 0.18 mmol) (7), methanesulfonyl chloride (0.02 mL, 0.21 mmol), DIPEA (0.06 mL, 0.35 mmol) and CH_2_Cl_2_ (2 mL). The reaction was stirred at room temperature for 16 h, diluted with CH_2_Cl_2_ and washed with brine. The organic layer was concentrated *in vacuo* and purified by column chromatography eluting with EtOAc in cyclohexane (0–100% gradient). The appropriate fractions were combined and concentrated *in vacuo* to give 4-(((3-(3,4-dimethoxyphenyl)-2,5-dimethylpyrazolo[1,5-*a*]pyrimidin-7-yl)amino)methyl)phenyl methanesulfonate 9 (82 mg, 0.17 mmol, 97% yield). ^1^H NMR (400 MHz, DMSO-d_6_) *δ* = 8.43 (s, 1H), 7.56–7.48 (m, 2H), 7.41 (d, *J* = 2.5 Hz, 1H), 7.37–7.29 (m, 2H), 7.22 (dd, *J* = 2.0, 8.4 Hz, 1H), 7.02 (d, *J* = 8.4 Hz, 1H), 6.04 (s, 1H), 4.68–4.59 (m, 2H), 3.80 (s, 3H), 3.79 (s, 3H), 3.36 (s, 3H), 2.55 (s, 3H), 2.34 (s, 3H). ^13^C NMR (151 MHz, DMSO-d_6_) *δ* = 159.1, 150.4, 148.9, 148.6, 147.3, 146.3, 138.2, 129.1, 126.5, 122.8, 121.0, 113.1, 112.6, 105.8, 85.9, 56.1, 56.0, 44.2, 37.9, 31.1, 25.3, 14.8. LCMS (formic); *t*_ret_ = 0.73 min, [M + H]^+^ 483.3. HRMS (C_24_H_26_N_4_O_5_S) [M + H]^+^ requires 483.1702 found [M + H]^+^ 483.1693.

### 5-(7-((4-Hydroxybenzyl)amino)-2,5-dimethylpyrazolo[1,5-*a*]pyrimidin-3-yl)-2-methoxyphenol (10)

A microwave vial (20 mL) was charged with 5-(2,5-dimethyl-7-phenoxypyrazolo[1,5-*a*]pyrimidin-3-yl)-2-methoxyphenol (520 mg, 1.44 mmol) (19), 4-(aminomethyl)phenol (266 mg, 2.16 mmol), DIPEA (0.503 mL, 2.88 mmol) and DMSO (11 mL). The reaction vessel was sealed and heated in a microwave at 130 °C for 5 h. After cooling, the reaction mixture was diluted with EtOAc and washed with brine. The organic layer was concentrated *in vacuo* and purified by column chromatography eluting with EtOAc in cyclohexane (0–100% gradient). The appropriate fractions were combined and concentrated *in vacuo* to 5-(7-((4-hydroxybenzyl)amino)-2,5-dimethylpyrazolo[1,5-*a*]pyrimidin-3-yl)-2-methoxyphenol 10 (459 mg, 1.18 mmol, 82% yield). ^1^H NMR (400 MHz, DMSO-d_6_) *δ* = 9.35 (s, 1H), 8.91 (s, 1H), 7.23 (d, *J* = 8.9 Hz, 2H), 7.16 (m, 1H), 7.04 (d, *J* = 2.0 Hz, 1H), 6.99–6.95 (m, 1H), 6.73 (d, *J* = 8.4 Hz, 2H), 6.02 (s, 1H), 4.48 (d, *J* = 6.4 Hz, 2H), 3.79 (s, 3H), 2.48 (s, 3H), 2.34 (s, 3H). ^13^C NMR (101 MHz, DMSO-d_6_) *δ* = 163.4, 157.1, 146.8, 129.0, 128.4, 120.1, 119.7, 116.6, 115.7, 113.0, 106.0, 86.3, 84.2, 71.6, 56.3, 44.6, 31.1, 21.2, 14.5 (one carbon signal not observed). LCMS (formic); *t*_ret_ = 0.6 min, [M + H]^+^ 391.3. HRMS (C_22_H_22_N_4_O_3_) [M + H]^+^ requires 391.1770 found [M + H]^+^ 391.1767.

### 4-(((3-(3-((Fluorosulfonyl)oxy)-4-methoxyphenyl)-2,5-dimethylpyrazolo[1,5-*a*]pyrimidin-7-yl)amino)methyl)phenyl sulfurofluoridate (11)

Chamber A was charged with sulfonyldiimidazole (104 mg, 0.52 mmol) (12) and potassium fluoride (81 mg, 1.39 mmol). Chamber B was charged with 5-(7-((4-hydroxybenzyl)amino)-2,5-dimethylpyrazolo[1,5-*a*]pyrimidin-3-yl)-2-methoxyphenol (68 mg, 0.17 mmol) (10), acetonitrile (3 mL) and DIPEA (0.73 mL, 4.18 mmol). Trifluoroacetic acid (2 mL) was added to chamber A *via* syringe injection and the reaction was stirred at room temperature for 16 h. Chamber B was concentrated *in vacuo*, diluted with CH_2_Cl_2_ and washed with brine. The organic layer was concentrated *in vacuo* and purified by MDAP (Method C). The appropriate fractions were combined and concentrated *in vacuo* to afford 4-(((3-(3-((fluorosulfonyl)oxy)-4-methoxyphenyl)-2,5-dimethylpyrazolo[1,5-a]pyrimidin-7-yl)amino)methyl)phenyl sulfurofluoridate 11 (25 mg, 0.05 mmol, 26% yield). ^1^H NMR (700 MHz, DMSO-d_6_) *δ* = 8.57 (t, *J* = 6.7 Hz, 1H), 7.98 (d, *J* = 2.1 Hz, 1H), 7.83 (dd, *J* = 2.1, 8.7 Hz, 1H), 7.62–7.59 (m, 2H), 7.59–7.54 (m, 2H), 7.41 (d, *J* = 8.9 Hz, 1H), 6.10 (s, 1H), 4.67 (d, *J* = 6.6 Hz, 2H), 3.94 (s, 3H), 2.57 (s, 3H), 2.34 (s, 3H). ^13^C NMR (176 MHz, DMSO-d_6_) *δ* = 159.3, 150.0, 148.6, 147.9, 146.0, 145.8, 139.7, 138.0, 129.3, 129.1, 126.7, 121.1, 121.0, 114.3, 103.2, 85.9, 56.4, 43.5, 24.8, 14.3. ^19^F NMR (500 MHz, DMSO-d_6_) *δ* = 40.31 (s, 1F), 38.43 (s, 1F). LCMS (formic) 99%; *t*_ret_ = 1.22 min, [M + H]^+^ 555.3. HRMS (C_22_H_20_F_2_N_4_O_7_S_2_) [M + H]^+^ requires 555.0820 found [M + H]^+^ 555.0818. *ν*_max_ (neat)/cm^−1^ 2928, 1711, 1618, 1580, 1442, 1290, 1228, 1140, 1018, 930, 840, 800.

## Conflicts of interest

There are no conflicts to declare.

## Supplementary Material

CB-004-D3CB00142C-s001

## References

[cit1] Mathers C. D., Loncar D. (2006). PLoS Med..

[cit2] Vijayan V. K. (2013). Indian J. Med. Res..

[cit3] Bestall J. C., Paul E. A., Garrod R., Garnham R., Jones P. W., Wedzicha J. A. (1999). Thorax.

[cit4] Morrow A. A., Alipour M. A., Bridges D., Yao Z., Saltiel A. R., Lee J. M. (2014). Mol. Cancer Res..

[cit5] Singh J., Petter R. C., Baillie T., Whitty A. (2011). Nat. Rev. Drug Discovery.

[cit6] Abranyi-Balogh P., Petri L., Imre T., Szijj P., Scarpino A., Hrast M., Mitrovic A., Fonovic U. P., Nemeth K., Barreteau H., Roper D. I., Horvati K., Ferenczy G. G., Kos J., Ilas J., Gobec S., Keseru G. M. (2018). Eur. J. Med. Chem..

[cit7] Ghosh A. K., Samanta I., Mondal A., Liu W. R. (2019). ChemMedChem.

[cit8] Hallenbeck K. K., Turner D. M., Renslo A. R., Arkin M. R. (2017). Curr. Top. Med. Chem..

[cit9] Lagoutte R., Patouret R., Winssinger N. (2017). Curr. Opin. Chem. Biol..

[cit10] Pan Z., Scheerens H., Li S. J., Schultz B. E., Sprengeler P. A., Burrill L. C., Mendonca R. V., Sweeney M. D., Scott K. C., Grothaus P. G., Jeffery D. A., Spoerke J. M., Honigberg L. A., Young P. R., Dalrymple S. A., Palmer J. T. (2007). ChemMedChem.

[cit11] Li D., Ambrogio L., Shimamura T., Kubo S., Takahashi M., Chirieac L. R., Padera R. F., Shapiro G. I., Baum A., Himmelsbach F., Rettig W. J., Meyerson M., Solca F., Greulich H., Wong K. K. (2008). Oncogene.

[cit12] Waterson A. G., Petrov K. G., Hornberger K. R., Hubbard R. D., Sammond D. M., Smith S. C., Dickson H. D., Caferro T. R., Hinkle K. W., Stevens K. L., Dickerson S. H., Rusnak D. W., Spehar G. M., Wood E. R., Griffin R. J., Uehling D. E. (2009). Bioorg. Med. Chem. Lett..

[cit13] Ponader S., Chen S. S., Buggy J. J., Balakrishnan K., Gandhi V., Wierda W. G., Keating M. J., O'Brien S., Chiorazzi N., Burger J. A. (2012). Blood.

[cit14] Cuesta A., Taunton J. (2019). Ann. Rev. Biochem..

[cit15] Mukherjee H., Grimster N. P. (2018). Curr. Opin. Chem. Biol..

[cit16] Barf T., Kaptein A. (2012). J. Med. Chem..

[cit17] Engel J., Lategahn J., Rauh D. (2016). ACS Med. Chem. Lett..

[cit18] Shannon D. A., Weerapana E. (2015). Curr. Opin. Chem. Biol..

[cit19] Platzer G., Okon M., McIntosh L. P. (2014). J. Biomol. NMR.

[cit20] Pettinger J., Jones K., Cheeseman M. D. (2017). Angew. Chem., Int. Ed..

[cit21] Powers J. C., Asgian J. L., Ekici O. D., James K. E. (2002). Chem. Rev..

[cit22] Hunkapiller M. W., Smallcombe S. H., Witaker D. R., Richards J. H. (1973). J. Biol. Chem..

[cit23] Harris T. K., Turner G. J. (2002). IUBMB Life.

[cit24] Hacker S. M., Backus K. M., Lazear M. R., Forli S., Correia B. E., Cravatt B. F. (2017). Nat. Chem..

[cit25] Liu R., Yue Z., Tsai C. C., Shen J. (2019). J. Am. Chem. Soc..

[cit26] Dalton S. E., Dittus L., Thomas D. A., Convery M. A., Nunes J., Bush J. T., Evans J. P., Werner T., Bantscheff M., Murphy J. A., Campos S. (2018). J. Am. Chem. Soc..

[cit27] London N., Miller R. M., Krishnan S., Uchida K., Irwin J. J., Eidam O., Gibold L., Cimermancic P., Bonnet R., Shoicheta B. K., Taunton J. (2014). Nat. Chem. Biol..

[cit28] Campos S., Dalton S. E. (2020). ChemBioChem.

[cit29] Fattah T. A., Saeed A., Albericio F. (2018). J. Fluorine Chem..

[cit30] Aatkar A., Vuorinen A., Longfield O. E., Gilbert K., Peltier-Heap R., Wagner C. D., Zappacosta F., Rittinger K., Chung C.-W., House D., Tomkinson N. C. O., Bush J. T. (2023). ACS Chem. Biol..

[cit31] Zhao Q., Ouyang X., Wan X., Gajiwala K. S., Kath J. C., Jones L. H., Burlingame A. L., Taunton J. (2017). J. Am. Chem. Soc..

[cit32] Mukherjee H., Debreczeni J., Breed J., Tentarelli S., Aquila B., Dowling J. E., Whitty A., Grimster N. P. (2017). Org. Biomol. Chem..

[cit33] Gilbert K. E., Vuorinen A., Aatkar A., Pogány P., Pettinger J., Grant E. K., Kirkpatrick J. M., Rittinger K., House D., Burley G. A., Bush J. T. (2023). ACS Chem. Biol..

[cit34] Dong J., Krasnova L., Finn M. G., Sharpless K. B. (2014). Angew. Chem., Int. Ed..

[cit35] Cramer R., Coffman D. (1961). J. Org. Chem..

[cit36] Veryser C., Demaerel J., Bieliunas V., Gilles P., De Borggraeve W. M. (2017). Org. Lett..

[cit37] Zhou H., Mukherjee P., Liu R., Evrard E., Wang D., Humphrey J. M., Butler T. W., Hoth L. R., Sperry J. B., Sakata S. K., Helal C. J., am Ende C. W. (2018). Org. Lett..

[cit38] Baranczak A., Liu Y., Connelly S., Du W. G., Greiner E. R., Genereux J. C., Wiseman R. L., Eisele Y. S., Bradbury N. C., Dong J., Noodleman L., Sharpless K. B., Wilson I. A., Encalada S. E., Kelly J. W. (2015). J. Am. Chem. Soc..

[cit39] Udompholkul P., Garza-Granados A., Alboreggia G., Baggio C., McGuire J., Pegan S. D., Pellecchia M. (2023). J. Med. Chem..

[cit40] Mejdrova I., Chalupska D., Kogler M., Sala M., Plackova P., Baumlova A., Hrebabecky H., Prochazkova E., Dejmek M., Guillon R., Strunin D., Weber J., Lee G., Birkus G., Mertlikova-Kaiserova H., Boura E., Nencka R. (2015). J. Med. Chem..

[cit41] Cosgrove B., Down K., Bertrand S., Tomkinson N. C. O., Barker M. D. (2021). Bioorg. Med. Chem. Lett..

[cit42] Strelow J. M. (2017). SLAS Discovery.

[cit43] Cravatt B. F., Wright A. T., Kozarich J. W. (2008). Annu. Rev. Biochem..

[cit44] Arcaro A., Wymann M. P. (1993). Biochem. J..

[cit45] Baggio C., Udompholkul P., Gambini L., Salem A. F., Jossart J., Perry J. J. P., Pellecchia M. (2019). J. Med. Chem..

[cit46] Charter N. W., Kauffman L., Singh R., Eglen R. M. (2006). J. Biomol. Screening.

[cit47] The PyMOL Molecular Graphics System, Version 2.0 Schrödinger, LLC

[cit48] Martin-Gago P., Olsen C. A. (2019). Angew. Chem., Int. Ed..

[cit49] Jones L. H. (2018). ACS Med. Chem. Lett..

[cit50] Boike L., Henning N. J., Nomura D. K. (2022). Nat. Rev. Drug Discovery.

[cit51] Zhou H., Mukherjee P., Liu R., Evrard E., Wang D., Humphrey J. M., Butler T. W., Hoth L. R., Sperry J. B., Sakata S. K., Helal C. J., am Ende C. W. (2018). Org. Lett..

[cit52] Catalano J. G., Gaitonde V., Beesu M., Leivers A. L., Shotwell J. B. (2015). Tetrahedron Lett..

[cit53] Chen X., Li H., Lin Q., Dai S., Yue S., Qu L., Li M., Guo M., Wei H., Li J., Jiang L., Xu G., Chen Y. (2022). Commun. Chem..

