# Supplementary material for: Covalent targeting of non-cysteine residues in PI4KIIIβ
Source: RSC Chem Biol. 2023 Oct 17;4(12):1111–22. doi: 10.1039/d3cb00142c (PMC10685791; doi:10.1039/d3cb00142c)
Supplement: CB-004-D3CB00142C-s001 [file CB-004-D3CB00142C-s001.pdf]

Electronic Supplementary Material (ESI) for RSC Chemical Biology.
This journal is © The Royal Society of Chemistry 2023

Supplementary Information for

Covalent targeting of non-cysteine residues in PI4KIIIβ

Brett Cosgrove,[a,b] Emma K. Grant,[a] Sophie Bertrand,[a] Kenneth D. Down,[a] Don O. Somers,[c] John Evans,[d] Nicholas C. O. Tomkinson[b,*] and Michael D. Barker[a,*]

[a]*Medicinal Chemistry, GlaxoSmithKline Medicines Research Centre, Stevenage, U.K.*
[b]*Department of Pure and Applied Chemistry, University of Strathclyde, Glasgow, U.K.*
[c]*Structural and Biophysical Science, GlaxoSmithKline Medicines Research Centre, Stevenage, U.K.*
[d]*Screening, Profiling and Mechanistic Biology, GlaxoSmithKline Medicines Research Centre, Stevenage, U.K.*

Contents

General Methods	S3
Physicochemical assays	S6
In vitro assays	S7
Protein Mass Spectrometry	S9
X-Ray crystallography	S11
Kinetics	S15
Sulfonyl Fluoride Derivative	S17
Copies of NMR Spectra	S18
References	S31

General methods

Solvents and reagents – Solvents and reagents were purchased from commercial suppliers and used as received unless otherwise noted.

Reaction monitoring – Reactions were monitored by either liquid chromatography-mass spectroscopy (LCMS) or thin-layer chromatography (TLC).

Thin-layer chromatography (TLC) – TLC was carried out using polyester-backed precoated silica plates (0.2 mm particle size). Spots were visualised under ultraviolet (UV) light of λ_{max} = 254 nm or 365 nm. Where spots were difficult to see, they were stained with either vanillin or phosphomolybdic acid before gentle drying.

Liquid chromatography mass spectrometry – LCMS analysis was completed on a Waters® Acquity UPLC instrument equipped with a BEH column (50 mm x 2.1 mm with 1.7 μm packing diameter) and a Waters® Micromass ZQ MS using alternate-scan positive and negative electrospray ionisation. Analytes were then detected as a summed UV wavelength spectrum between 210-350 nm.

Two methods were used:

Formic: 40 °C, 1 mL/min flow rate, using a gradient with mobile phases of water containing 0.1% volume (*v/v*) of formic acid and MeCN with 0.1% formic acid (*v/v*). Gradient conditions were initially 1% of the MeCN mixture, increasing linearly to 97% over 1.5 min, and remaining at 97% for 0.4 min, before increasing to 100% over 0.1 min.

High pH: 40 °C, 1 mL/min flow rate, using a gradient of mobile phases of 10.0 mM aqueous ammonium bicarbonate solution, adjusted to pH 10.0 with 0.880 M aqueous ammonia and MeCN. Gradient conditions began at 1% MeCN, and increased linearly to 97% MeCN over 1.5 min, before remaining at this concentration for 0.40 min, and rising to 100% over 0.1 min.

Silica gel column chromatography – Column chromatography was carried out using the Teledyne ISCO Combi*Flash*® R_f + apparatus with Redi*Sep*® silica cartridges.

Nuclear magnetic resonance (NMR) spectroscopy – Proton (^{1}H) and carbon (^{13}C) spectra were recorded in deuterated solvents at ambient temperatures (unless otherwise stated) using the standardised pulse methods on the Bruker AV-400 (^{1}H = 400 MHz, ^{13}C = 101 MHz) spectrometer. Chemical shifts are reported in ppm and referenced to tetramethylsilane (TMS) or the following solvent peaks: $CDCl_3$ (^{1}H = 7.27 ppm, ^{13}C = 77.0 ppm), d_4-Methanol

(^{1}H = 3.31, ^{13}C = 49.0) or d_6-DMSO (^{1}H = 2.50 ppm, ^{13}C = 39.5 ppm). Peak assignments were chosen based on chemical shifts, integrations, and coupling constants, considering 2D analyses where necessary. Coupling constants are quoted to the nearest 0.1 Hz and multiplicities described as singlet (s), doublet (d), triplet (t), quartet (q), quintet (quin), sextet (sxt), septet (sept), broad (br.) or multiplet (m).

High-resolution mass spectrometry – High-resolution mass spectra were recorded on a micromass Q-Tof Ultima® time-of-flight mass spectrometer, and analytes were separated on an Agilent® 1100 Liquid Chromatograph equipped with a Penomenex® Luna C18 (2) reversed phase column (100 mm x 2.1 mm, 3 µm packing diameter). Conditions used were 0.5 mL/min flow rate at 35 °C, and an injection volume of 2–5 µL, using a gradient elution made up of water with 0.1% volume formic acid (*v/v*) and 0.1% formic acid (*v/v*) in MeCN. Elution conditions began with 5% MeCN mixture, and increased linearly to 100% over 6 min, before remaining at 100% for 2.5 min, decreasing back to 5% MeCN mixture for 1 min and equilibrating for 2.5 min prior to the next injection in the machine. Mass to charge (*m/z*) ratios are shown in Daltons.

Mass directed auto preparation (MDAP) chromatography – Mass directed auto preparation was carried on a Waters® ZQ MS using alternate scan positive and negative electrospray ionisation and a summed UV wavelength of 210–350 nm. According to the following methods:

Compound **3** and **4** (Method A)

Column: Xselect CSH C18 column (150 mm x 30mm i.d. 5 µm packing diameter)
Solvent A: Water (0.1% TFA)
Solvent B: Acetonitrile (0.1% TFA)
Gradient: From 15 to 45% (24.5 min).
Flow: 30 mL/min

Compound **8** (Method B)

Column: Xselect CSH C18 column (150 mm x 30 mm i.d. 5 µm packing diameter)
Solvent A: Water (0.1% Formic acid)
Solvent B: Acetonitrile (0.1% Formic acid)
Gradient: From 15 to 55% (24.5 min).
Flow: 40 mL/min

Compound **11** (Method C)

Column: Xselect CSH C18 column (150 mm x 30 mm i.d. 5 µm packing diameter)
Solvent A: Water (0.1% Formic acid)
Solvent B: Acetonitrile (0.1% Formic acid)
Gradient: From 30 to 85% (24.5 min)
Flow: 40 mL/min

Compound **15** (Method D)

Column: Xselect CSH C18 column (150mm x 30mm i.d. 5µm packing diameter)
Solvent A: 10mM ammonium bicarbonate adjusted to pH 10 with ammonia in water
Solvent B: Acetonitrile
Gradient: From 50 to 99% (20.5 min)
Flow: 40 ml/min

Compound **16** (Method E)

Column: Xselect CSH C18 column (150mm x 30mm i.d. 5µm packing diameter)
Solvent A: 10mM ammonium bicarbonate adjusted to pH 10 with ammonia in water
Solvent B: Acetonitrile
Gradient: From 15 to 55% (10 min)
Flow: 40 ml/min

Microwave – Reactions were carried out in a Biotage Initiator microwave, model: INITIATOR ROBOT SIXTY 355381,20233-14T.

Physicochemical Assays

Kinetic solubility – The kinetic aqueous solubility at pH 7.4 was determined by measuring the concentration of solute in solution after precipitation from DMSO stock solution. The DMSO stock solution was diluted 20-*fold* with PBS pH 7.4 and the solubility of the compound is measured after 1 h equilibration at room temperature. In this procedure quantification was done using a Charged Aerosol Detector (CAD). The DMSO concentration of the stock solution was also determined using this technique. Calibration parameters generated for CAD response of two calibrants (Primidone and Ketoconazole) are used to calculate the solubility of solutes considering the density of the compound and ion-pairing effects.

Artificial Membrane Permeability assay

The permeability rate was measured across a phospholipid bilayer system. The lipid was egg phosphatidyl choline (1.8%) and cholesterol (1%) dissolved in *n*-decane. This was applied to the bottom of the microfiltration filter inserts in a Transwell plate. Phosphate buffer (50 mM Na_2HPO_4 with 0.5% 2-hydroxypropyl-β-cyclodextrin), pH 7.4 was added to the top and bottom of the plate. The lipids were allowed to form bilayers across the small holes in the filter. Permeation experiment was initiated by adding the compound to the bottom well and stopped at a pre-determined elapsed time. The compound permeated through the membrane to enter the acceptor well. The compound concentration in both the donor and acceptor compartments was determined by liquid chromatography after 3 h incubation at room temperature. The permeability ($logP_{app}$) measured how fast molecules pass through the black lipid membrane was expressed in nm/s.

Lipophilicity: ChromlogD assay

The Chromatographic Hydrophobicity Index (CHI) values are measured using reversed phase HPLC column (50 x 2 mm 3 µM Gemini NX C18, Phenomenex, UK) with fast acetonitrile gradient at starting mobile phase of pH 2, 7.4 and 10.5. CHI values were derived directly from the gradient retention times using calibration parameters for standard compounds. The CHI value approximates to the volume % organic concentration when the compound elutes. CHI was linearly transformed into ChromlogD by least-square fitting of experimental CHI values to calculated ClogP values for over 20K research compounds using the following formula: ChromlogD = 0.0857CHI-2.00.

In Vitro Assays

Protein supply for biochemical assays and mass spectrometry studies

Human PI4KIIIβ (residues 13-828, 316-330 deleted) was expressed with a 6H-TEV n-terminal tag by baculovirus in insect cells. The resulting lysate was purified by nickel affinity and hydroxyapatite chromatography. The tag was removed by TEV-cleavage, followed by nickel affinity chromatography and size exclusion chromatography to provide the purified protein at 1 mg/mL in 50 mM Tris-HCl pH7.5, 250 mM NaCl, 5 mM TCEP, 1 mM CHAPS, 10% Glycerol.

Enzymatic assay (PI4KIIIβ pIC$_{50}$) – Compound plates stamped with 60 nL stamp from 11-point titration (10 µM final assay concentration), 60 nL of DMSO in control columns 6 and 18. Dispense 3 µL of 2x substrate solution into all columns and add 3 µL of assay buffer (25 mM HEPES pH 7.5 (NaOH), 10 mM MgCl, 0.5 mM EGTA, 0.1% Triton X-100, 2 mM TCEP, 0.1 mg/mL BSA, 100 mM ATP (fresh)) to column 18 (no enzyme low control). Add 3 µL of 2x enzyme solution to columns 1–17 and 19–24 and centrifuge plates at 1000 rpm for 1 min, apply plate seal to each plate and incubate for 180 min at room temperature. Centrifuge plates at 1000 rpm for 1 min prior to removal of plate seal and add 6 µL of ADP-Glo 1 reagent +0.1% CHAPS to all wells. Centrifuge plates at 1000 rpm for 1 min and incubate for 60 min at room temperature. Add 12 µL of ADP-Glo 2 reagent +0.1% CHAPS to all wells, centrifuge plates at 1000 rpm for 1 min and incubate for 40 min at room temperature. Read plates on Viewlux or PHERAstar (signal stable for 30–90 min following Glo 2 incubation). In the enzymatic assay, the ADP product is detected through a 2-step coupled detection with a luminescent read-out. In the first step the kinase reaction is quenched, and unused ATP substrate is converted to AMP, removing it from the detection system. In the second step, the ADP produced by the kinase reaction is converted back to ATP, and this is then detected *via* a luciferin/luciferase reaction, as shown in **Figure S1**. The luminescent signal produced is proportional to the quantity of ADP produced, therefore correlating with PI4KIIIβ activity.

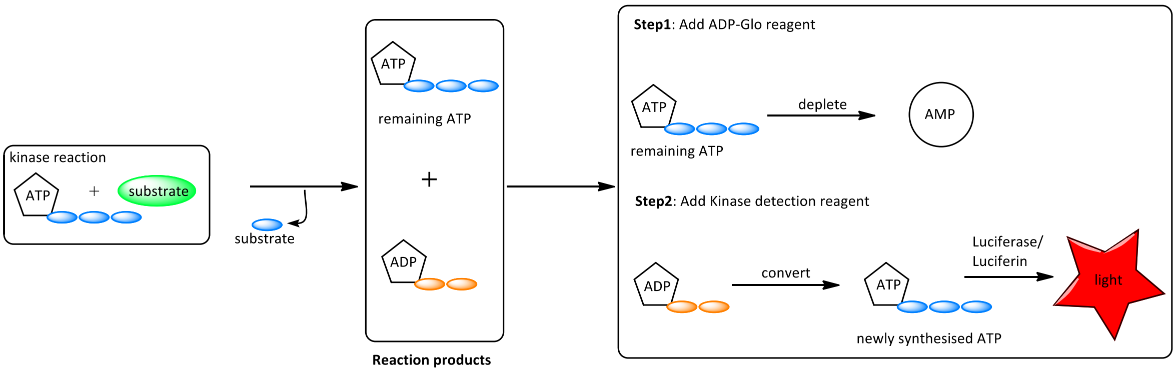

Figure S1: PI4KIIIβ enzyme assay.

Cellular based assay (CPE pIC$_{50}$) – 0.5 µL of a 10 mM compound stock solution is stamped into compound plates. 3 mM top conc, 1:3 dilutions, 10 points with 0.5 µL of 100% DMSO in columns 11 and 12. Remove the compound plates from the freezer, if necessary, and equilibrate to room temperature. Centrifuge briefly (bring up to 1000 rpm and then turn off the centrifuge). Remove the foil seals and pre-warm assay medium to 37 °C in sterile water bath. Wash confluent monolayer of HeLa cells with 10 mL of PBS and add 3 mL TryplE to cover cells. Incubate 5 min at 37 °C and gently agitate flask to detach cells, then remove cells to 50 mL Falcon tube. Centrifuge 300 g for 5 min and re-suspend pellet in approx. 50 mL of media, then count the cells using Vicell. Adjust concentration to 6.6 x 10^4 cells/mL, for the 96-well assay, cells and virus are pre-mixed prior to dispensing into the compound plates. Add virus to moi 1 and HRV type A strain 16 stock, or a pre-diluted stock, is added to the 6.6 x 10^4 cells/mL suspension at the appropriate pre-determined dilution for the current virus stock to achieve an MOI of 1, or a suitable MOI for the current virus stock. Add 150 µL of cell/virus suspension per well using a Multidrop to columns 1 to 11 and add 150 µL of cell suspension per well using a multichannel pipette to column 12. Incubate the plates at 33 °C, 5% CO_2, for 2 days post infection. Prepare fresh, or thaw frozen, CellTiter-Glo reagent. Avoid repeated freeze thaw cycles. Remove the 96-well plates from the 33 °C, 5% CO_2 incubator and equilibrate to room temperature. Dispense 60 µL CellTiter-Glo reagent into all columns of the 96-well plates using a Multidrop. Cell Titer Glo reagent can be stored at RT or 4 °C for 8 h. Once reconstituted can be stored at -20 °C for 21 weeks with ~3% loss of activity. Stable up to 10 freeze thaw cycles with <10% loss of activity. After approximately 20 minutes of room temperature incubation, read the 96-well plates on the Envision using the Cell Titre Glo for CPE protocol to measure luminescence.

Protein Mass Spectrometry

Intact protein mass spectrometry – Intact protein masses were recorded by liquid chromatography-mass spectrometry (LC-MS) using a 6224 TOF (Agilent) Accurate Mass Series mass spectrometer, interfaced with an Agilent 1200 liquid chromatography and sample handling system. The protein sample was injected using an Agilent 1200 series AutoSampler (Model No. G1367B) with a 10 µL injection volume and maintained at a temperature of 10 °C. Chromatography was carried out on an Agilent Bio-HPLC PLRP-S (1000 Å, 5 µm × 50 mm × 1.0 mm, PL1312-1502) reverse phase HPLC column at 70 °C. Using an Agilent 1200 series binary pump system (Model No. G1312B) the sample was eluted at 0.5 mL/min using a gradient system from Solvent A (water, 0.2% (*v/v*) formic acid) to Solvent B (MeCN, 0.2% (*v/v*) formic acid) according to the following conditions:

TIME (MIN)	%B	FLOW (ML/MIN)	PRESSURE
0	20	0.5	350
0.5	20	0.5	350
0.51	40	0.5	350
2.5	80	0.5	350
2.51	100	0.5	350
4	100	0.5	350
4.01	20	0.5	350
4.5	20	0.5	350

The eluent was injected directly into an Agilent TOF mass spectrometer (Model No. G6224A) using a dual ESI source and scanning between 600–3200 Da with a scan rate of 1.03 s in positive mode. The following MS parameters were used: Capillary voltage limit – 4200; Desolvation temperature – 340 °C; Drying gas flow – 8.0 L/min. Data acquisition was carried out in 2 GHz Extended Dynamic range mode. Spectra were processed using Mass Hunter Qualitative Analysis™ B06.00 (Agilent) software with the Maximum Entropy method employed. The total ion chromatograms (TIC) were extracted (region containing protein) and the summed scans were deconvoluted over a *m/z* range with an expected mass range dependent on the protein (see below).

Deconvolution conditions for PI4KIIIβ.

PROTEIN	M/Z RANGE	EXPECTED MASS RANGE
PI4KIIIβ	850–2200	89000–93000

The .csv files of deconvoluted spectra results were analysed using R Studio software to generate .TIFF files of the spectra.

X-Ray Crystallography

Method and statistics: Human PI4KIIIβ (residues 291-801, 416-503 deleted, R409Q, R412Q) was expressed with a cleavable GST n-terminal tag by baculovirus in Sf9 insect cells. The tag was removed by TEV-cleavage after cell lysis, followed by nickel affinity chromatography and size exclusion chromatography. The purified protein at 7.5 mg/mL (in 20 mM Tris-HCl pH 7.5, 200 mM NaCl, 5 mM TCEP, 5% glycerol, 5 μg/mL Leupeptin) was combined with compound (2 mM) and $MgCl_2$ (2 mM), incubated for ~4 h on ice and subsequently crystallized by vapour diffusion over well buffer (2.4–2.7 M Sodium Formate pH 7) at 20°C. The drop comprised the protein + compound solution (1.5 μL) with well buffer (1 μL) and seedstock (0.5 μL). The seedstock contained seeds stabilized in approximately 3.4 M Sodium Formate pH 7. Cocrystals were cryoprotected with 2.8 M Sodium Formate pH 7, 5 mM $MgCl_2$, 25% ethylene glycol, supplemented with up to 5 mM compound, prior to cryofreezing in liquid nitrogen and obtaining synchrotron X-ray diffraction data, at 100 K.

The diffraction data were processed using AUTOPROC[1] with STARANISO,[2] which also employ XDS,[3] POINTLESS,[4] AIMLESS[5] and CCP4[6] at different stages. All complexes were solved by Fourier Synthesis using PI4KIIIb protein coordinates determined in-house. Structure refinements were carried out using REFMAC[7] and model building with COOT,[8] The final R_{factor} (and R_{free}) achieved for compounds **4**, **8** and **11** crystal structures are shown in Table S1, with associated PDB deposition codes.

Table S1:

**Compound**	**4**	**8**	**11**
**Data collection and processing**			
Beamline	DLS (I041)	ESRF (ID30A-1)	DLS (I04)
Wavelength (Å)	0.91587	0.96600	0.97950
Diffraction limits (Å)	2.042 1.506 1.530	2.576 1.639 1.541	2.895 1.978 1.871
Resolution range (Å)	57.11 - 1.51 (1.62 - 1.51)	57.29 - 1.54 (1.71 - 1.54)	57.30 - 1.94 (2.09 - 1.94)
Space group	P 21 21 21	P 21 21 21	P 21 21 21
Unit cell	59.211 67.553 106.932 90 90 90	57.737 67.838 106.947 90 90 90	58.839 67.845 107.033 90 90 90
Total reflections	204421 (8408)	166199 (9535)	135413 (6606)
Unique reflections	43748 (2187)	33639 (1682)	20831 (1042)
Multiplicity	4.7 (3.8)	4.9 (5.7)	6.5 (6.3)
Completeness – ellipsoidal (%)	87.2 (64.5)	93.6 (68.9)	93.9 (64.0)
Mean I/sigma(I)	16.5 (1.6)	8.4 (1.6)	10.0 (1.3)
Wilson B-factor	30.11	26.57	33.02
R-merge	0.039 (0.647)	0.095 (1.119)	0.134 (1.464)
R-meas	0.044 (0.754)	0.106 (1.233)	0.146 (1.598)
CC1/2	0.999 (0.766)	0.997 (0.605)	0.997 (0.516)
**Refinement**			
Reflections used in refinement	41521	31937	19774
Reflections used for R-free	2188	1663	1016
R-work	0.1792	0.1673	0.1695
R-free	0.2239	0.2213	0.2368
**Number of non-hydrogen atoms**	3631	3577	3420
macromolecule	3221	3129	3131
compound	31	33	35
Solvent (Water)	344	402	249
Solvent (Others)	35	13	5
Protein residues	389	385	400
RMS (bonds) [Å]	0.004	0.004	0.004
RMS (angles) [deg]	1.107	1.021	1.111
**Average B-factor**	31.99	26.00	35.00
macromolecules	30.89	24.74	34.78
ligands	27.14	23.65	39.34
Solvent (Waters)	41.34	35.43	37.18
Solvent (non-Waters)	45.70	44.05	34.97
**PDB code**	8Q6F	8Q6G	8Q6H

Ramachandran plots for each structure:

Ramachandran plots for each crystal structure. The key below the plot details what type of residue each point represents, and each point on the plot is coloured green for favoured, yellow for allowed and red for an outlier. The residue type and position of each outlier is indicated in blue using the three-letter residue code. (Images produced by PSILO[9]).

Compound **4**:

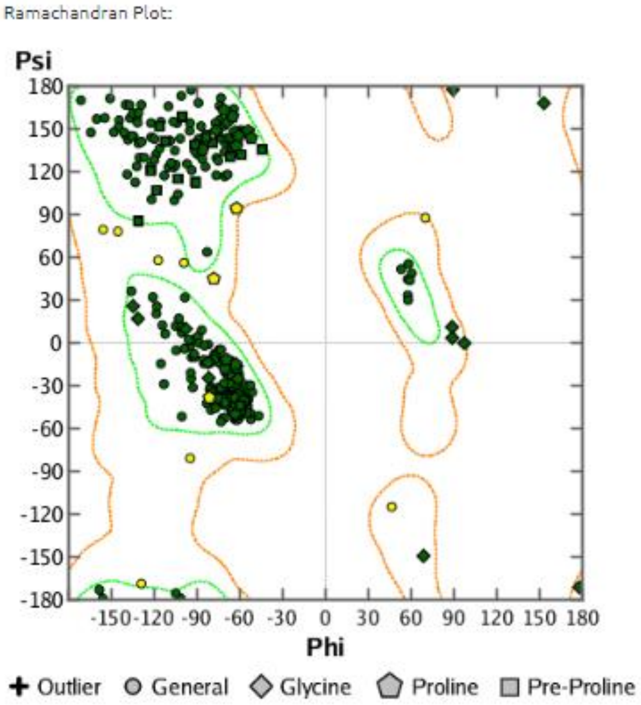

Compound **8**:

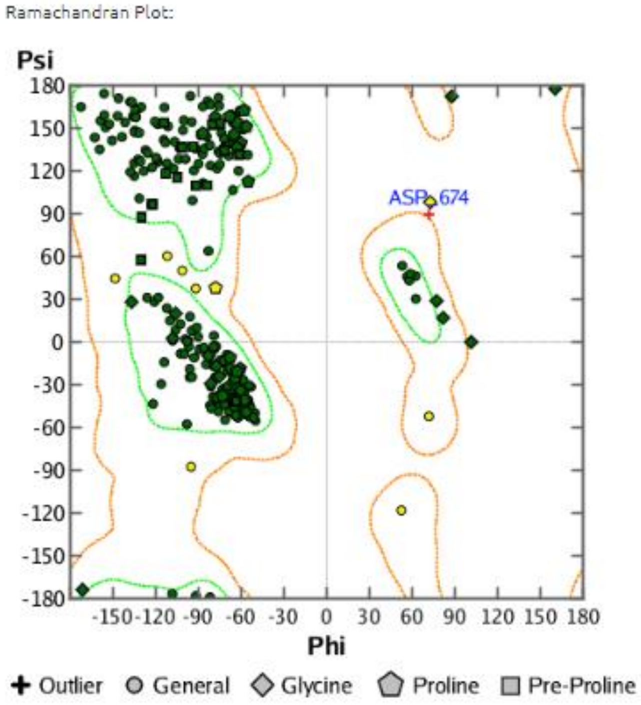

Compound **11**:

Ramachandran Plot:

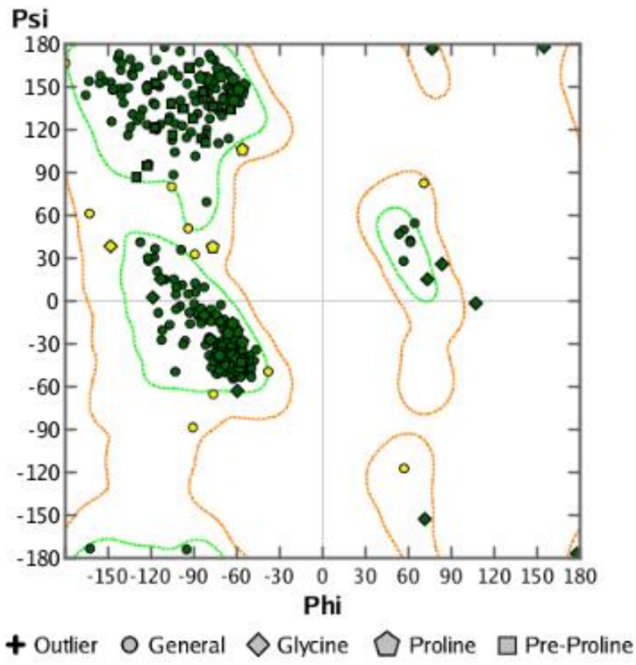

Kinetic Binding Assay

Human PI4KIIIβ (13-828, D316-330): Stored at -80 °C. No known stability issues but should avoid repeated freeze/thaw cycles (>3). Molecular Weight: approx. 90,292 Da. The total protein concentration is approx. 1 mg/mL (Bradford; equivalent to 11.1 μM). TCEP: Stored at -20 °C in reasonably sized aliquots. Bovine Serum Albumin (BSA) (Molecular Weight approx. 66,000 Da). Prepare 10 mg/mL in MilliQ water; stored at -20 °C in reasonably sized aliquots. Phosphatidylinositol (diC8), PI (Molecular Weight 608.6 Da). Dilute to 5 mM in MilliQ water and store at -80 °C in reasonably sized aliquots. ATP (10 mM). ADP-Glo kit, 0.1% CHAPS added to Glo1 and Glo2 reagents, stored at -20 °C. Buffered solution (contains 25 mM HEPES pH 7.5 (NaOH), 0.5 mM EGTA, 10 mM $MgCl_2$, 0.1% (*v/v*) Triton X-100). Add 2 mM TCEP and 0.1 mg/mL BSA on day of use. Compound stock solutions (10 mM) were dispensed using the HP digital dispenser using diluted stocks. The total final volume of DMSO added to each well was normalized across the plate to 60.8 nL (1%) in a total volume of 6 µL. White Greiner 384 well low volume plates were used. The onset of inhibition at saturating ATP (1 mM to exacerbate slow association) using the ADP-Glo format. The substrate, enzyme, and quench additions were performed by the DragonFly liquid handling robot. Global progress curve fitting was used to determine the rate of onset of inhibition at increasing concentrations of inhibitor. The raw fluorescence data was fitted by the local progress curves analysis assuming a slow binding, irreversible inhibition. The notation for use of this equation in Graphpad Prism 5 is given below. Where k_{inact} denotes the rate constant for inactivation of the kinase in units of s^{-1}, and K_i is the concentration at which the half maximal rate of inactivation is achieved, in molar units to derive the k_{inact}. K_i values are an expression of an IC_{50} value as described by the Cheng-Prusoff relationship therefore k_{on}/k_{off} describes the change in K_i. [s] is the concentration of substrate used. K_m is the Michaelis Menton constant, substrate concentration at which 50% of the maximal enzyme turnover rate is achieved. Therefore, for assays carried out at K_m(ATP), the K_i is approximately 2-*fold* more potent than the measured value, which corresponds to roughly 0.3 log unit increase in pIC_{50}.

$$\text{Cheng Prusoff equation: } K_i = \frac{Ic_{50}}{1 + \left(\frac{[s]}{K_m}\right)}$$

GraphPad notation:

vf = 0

vi = v0 /(1 + (Compound*1e-9)/ K_i)

k_{obs} = ((k_{inact} * (Compound*1e-9))/(K_i + (Compound*1e-9)))

Y=If(X<X0, Y0, Y0+ vf*(X-X0) + ((vi-vf)/k_{obs})*(1-exp(-k_{obs}*(X-X0))))

Sulfonyl Fluoride Derivative

In the early stages of this investigation the sulfonyl fluoride **S1** was prepared prior to examining fluorosulfate ligands.

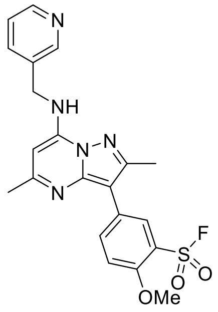

S1

Whilst the literature suggests that aryl sulfonyl fluorides should be stable, on this template, **S1** proved susceptible to rapid hydrolysis and the corresponding sulfonic acid was observed by LCMS. As the results obtained from using this warhead proved unconvincing we turned our attention to the fluorosulfate series.

Copies of NMR Spectra

2-Chloro-5-(2,5-dimethyl-7-((pyridin-4-ylmethyl)amino)pyrazolo[1,5-a]pyrimidin-3-yl)phenyl sulfofluoridate **3**: ^{1}**H NMR** (400 MHz, DMSO-d$_6$)

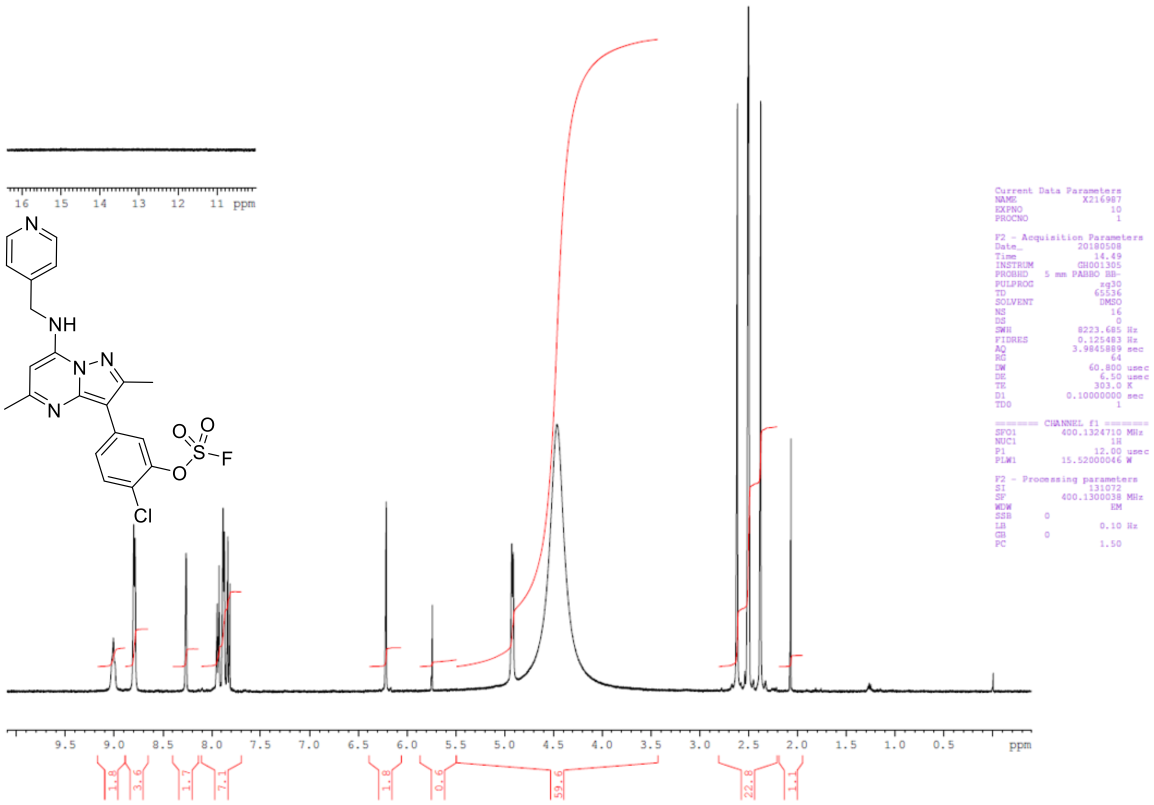

2-Chloro-5-(2,5-dimethyl-7-((pyridin-4-ylmethyl)amino)pyrazolo[1,5-a]pyrimidin-3-yl)phenyl sulfofluoridate **3**: ^{13}**C NMR** (DMSO-d$_6$, 151 MHz)

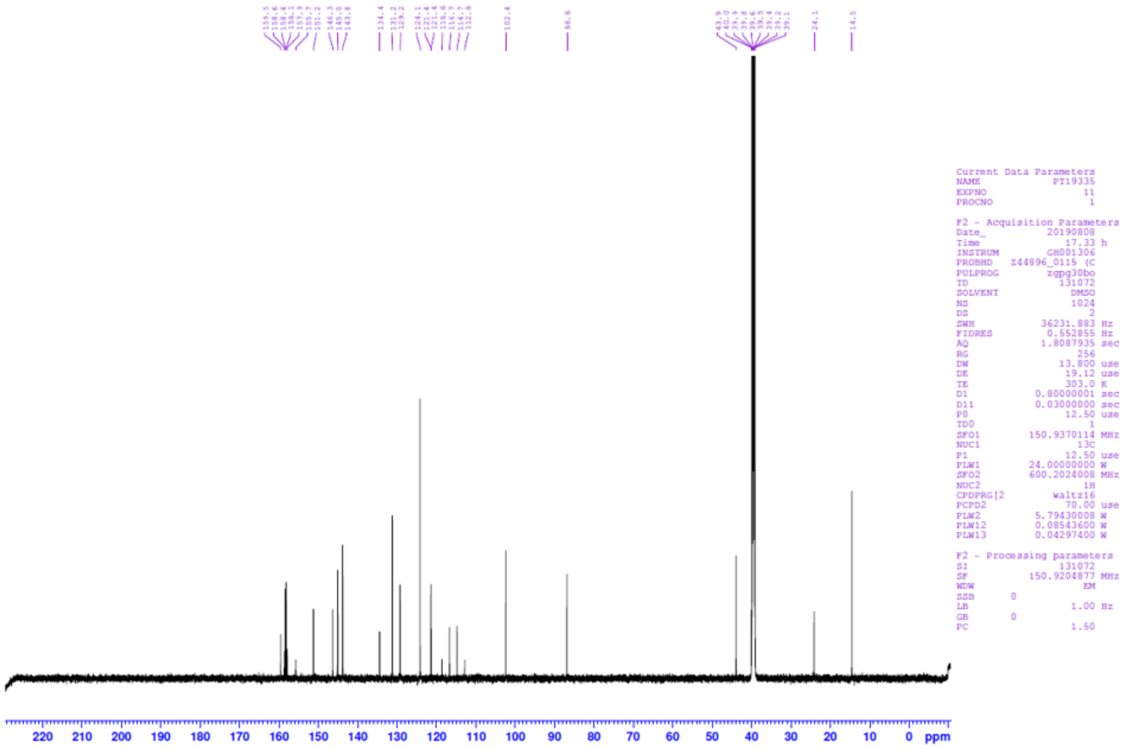

5-(2,5-Dimethyl-7-((pyridin-4-ylmethyl)amino)pyrazolo[1,5-*a*]pyrimidin-3-yl)-2-methoxyphenyl sulfofluoridate **4**: **^{1}H NMR** (400 MHz, DMSO-d$_6$)

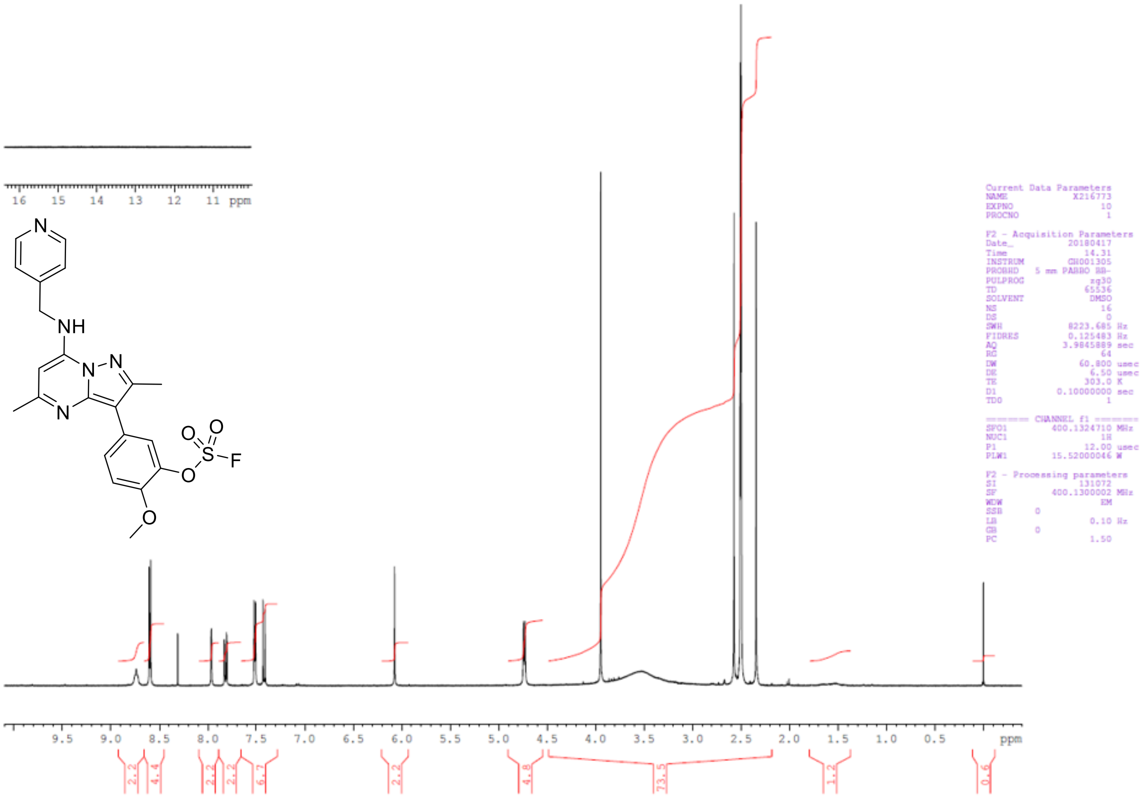

5-(2,5-Dimethyl-7-((pyridin-4-ylmethyl)amino)pyrazolo[1,5-*a*]pyrimidin-3-yl)-2-methoxyphenyl sulfofluoridate **4**: **^{13}C NMR** (101 MHz, DMSO-d$_6$)

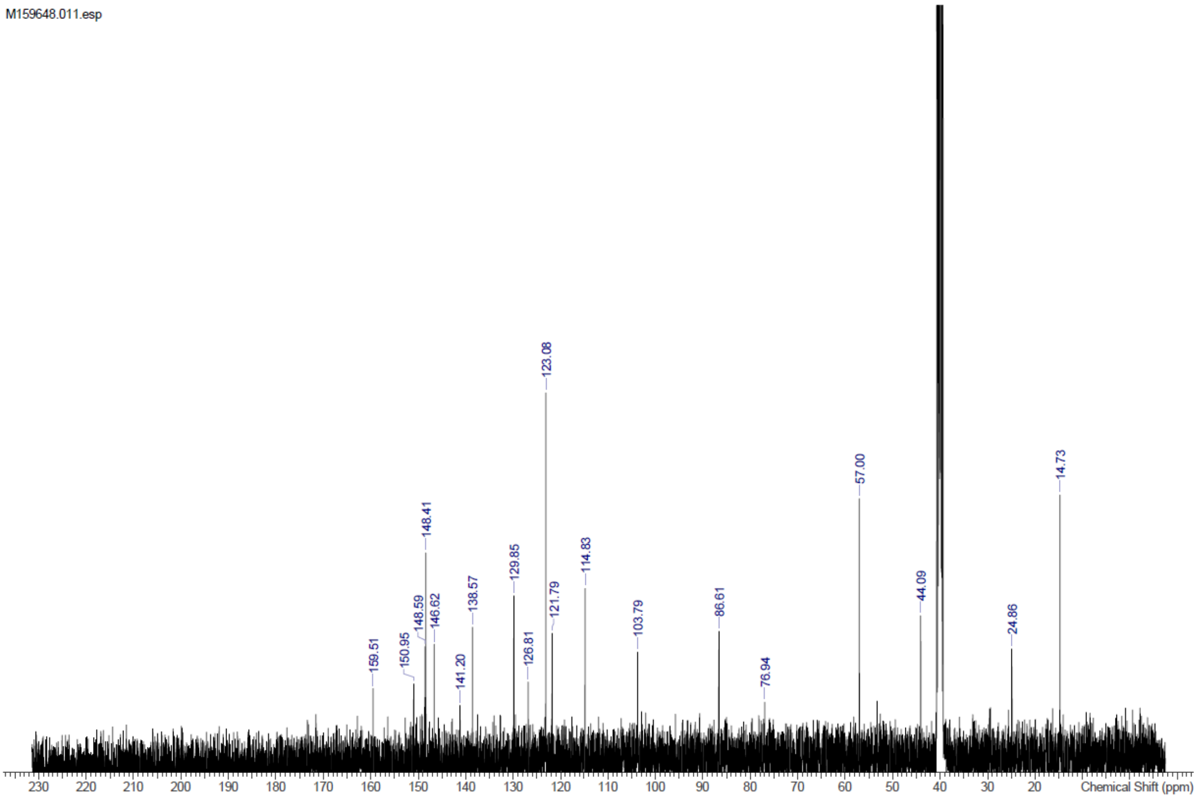

5-(2,5-Dimethyl-7-((pyridin-4-ylmethyl)amino)pyrazolo[1,5-*a*]pyrimidin-3-yl)-2-methoxyphenyl sulfofluoridate **4**:**^{19}F NMR** (376 MHz, DMSO-d6)

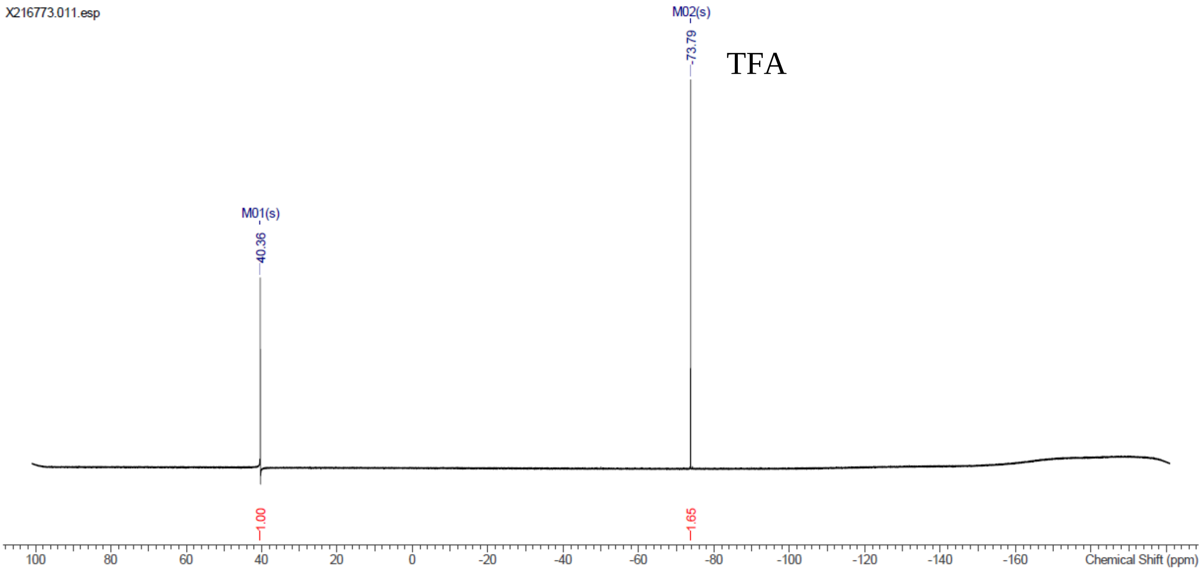

5-(2,5-Dimethyl-7-((pyridin-4-ylmethyl)amino)pyrazolo[1,5-a]pyrimidin-3-yl)-2-methoxyphenol **5**: **^{1}H NMR** (400 MHz, DMSO-d$_6$)

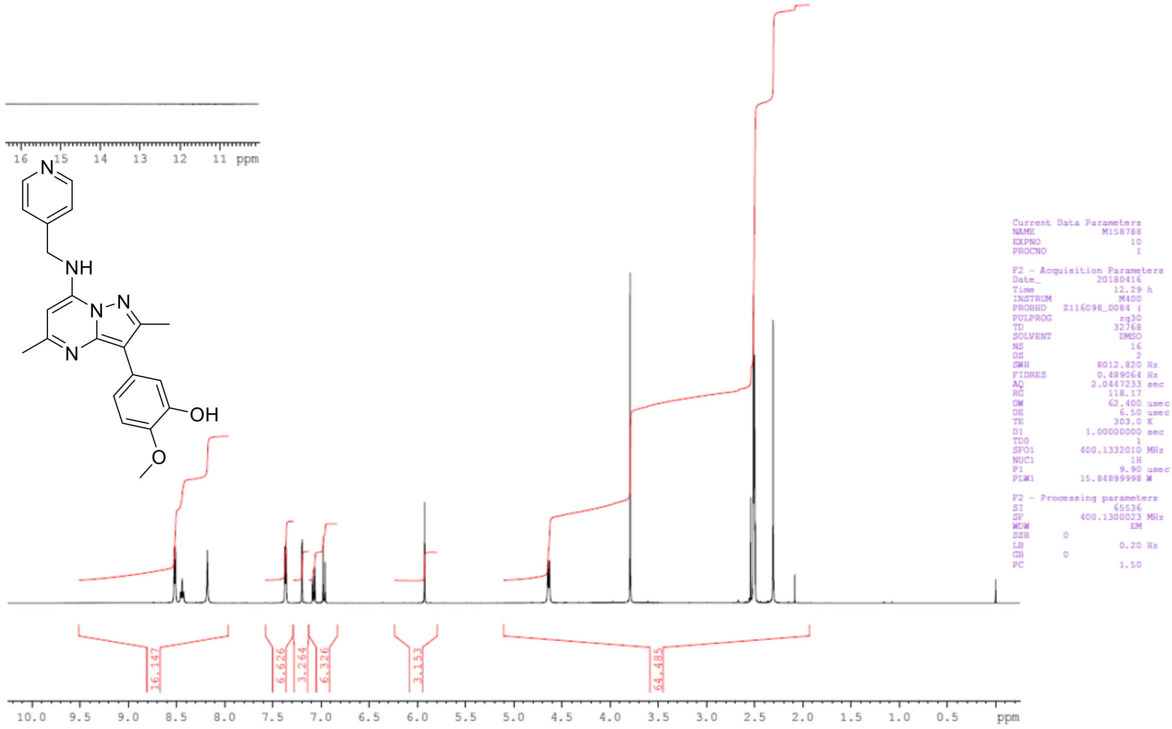

5-(2,5-Dimethyl-7-((pyridin-4-ylmethyl)amino)pyrazolo[1,5-a]pyrimidin-3-yl)-2-methoxyphenol **5**: **^{13}C NMR** (DMSO-d$_6$, 101 MHz)

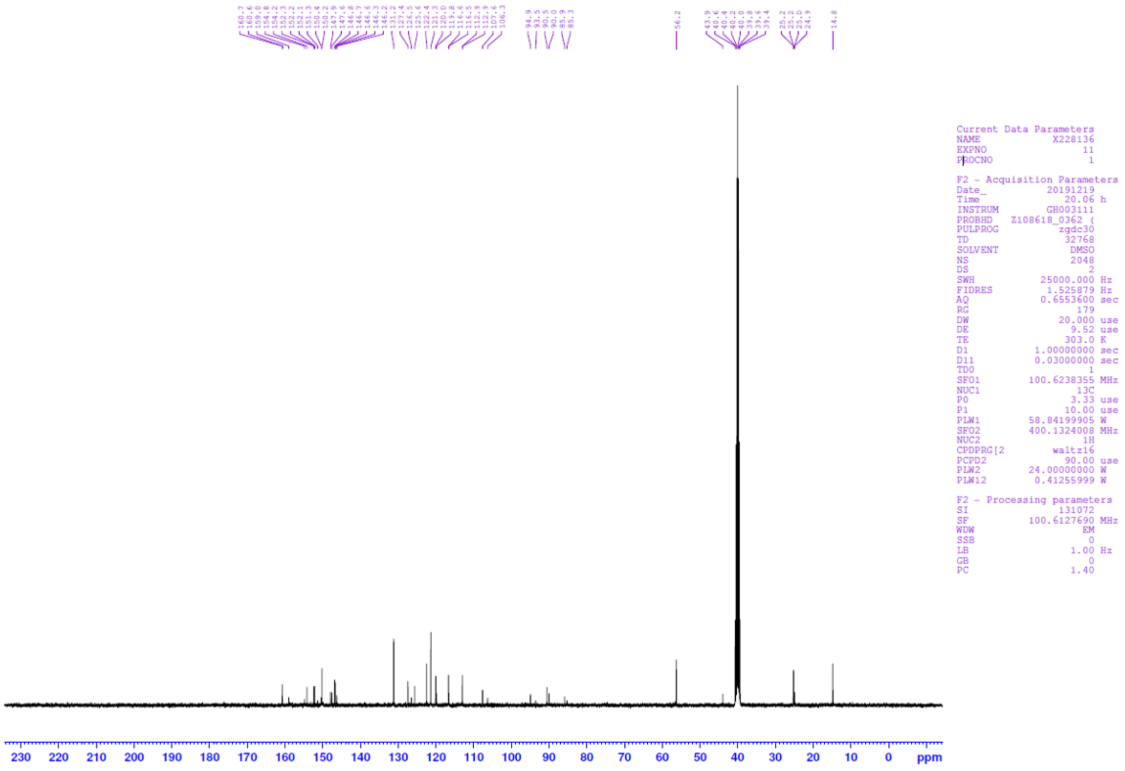

5-(2,5-Dimethyl-7-((pyridin-4-ylmethyl)amino)pyrazolo[1,5-a]pyrimidin-3-yl)-2-methoxyphenyl methanesulfonate **6**: **^{1}H NMR** (400 MHz, DMSO-d$_6$)

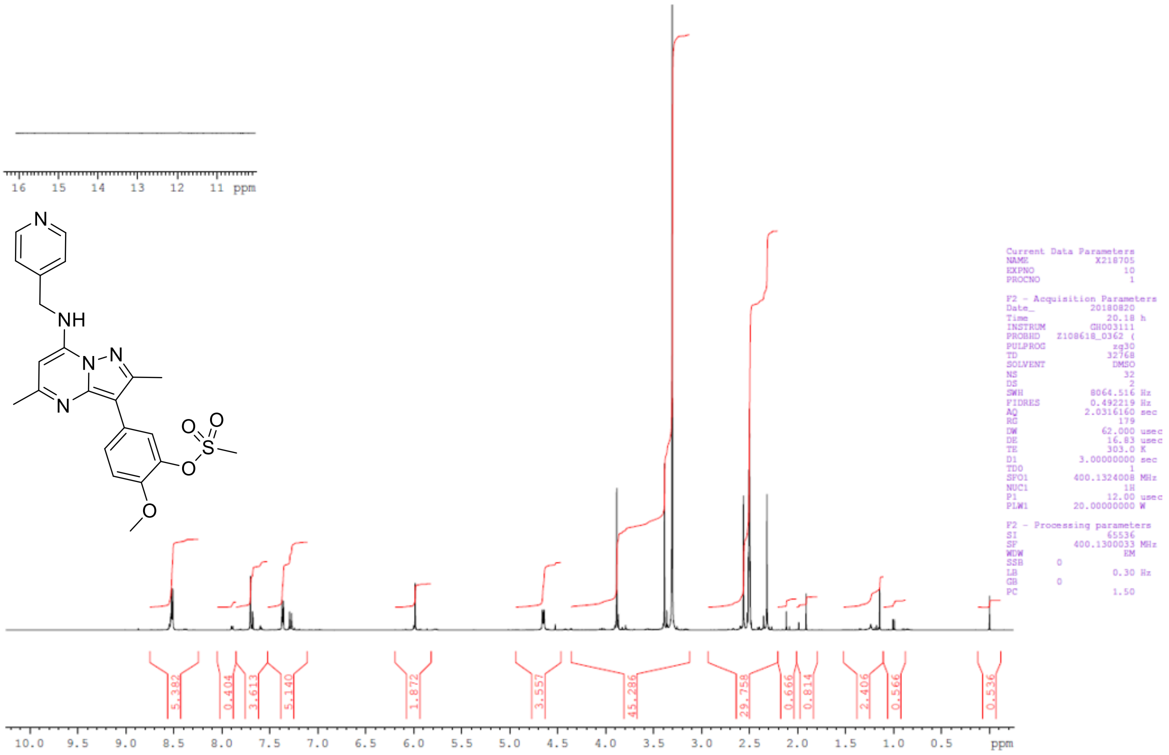

5-(2,5-Dimethyl-7-((pyridin-4-ylmethyl)amino)pyrazolo[1,5-a]pyrimidin-3-yl)-2-methoxyphenyl methanesulfonate **6**: **^{13}C NMR** (101 MHz, DMSO-d$_6$)

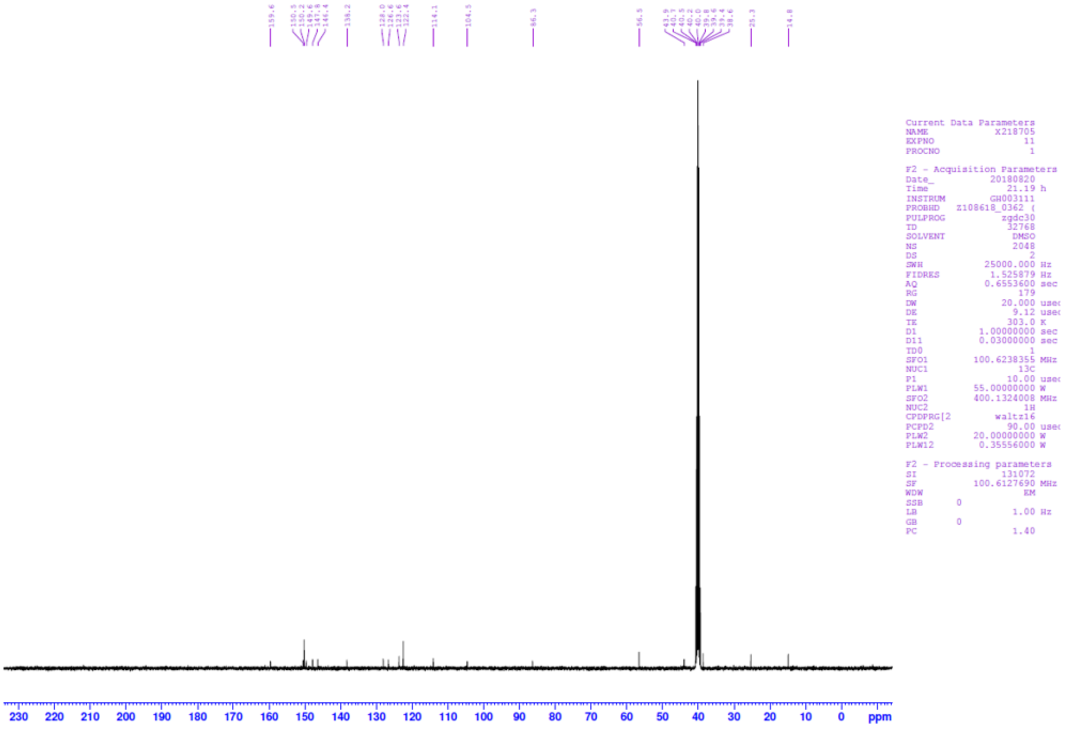

4-(((3-(3,4-Dimethoxyphenyl)-2,5-dimethylpyrazolo[1,5-a]pyrimidin-7-yl)amino)methyl)phenol **7**: **^{1}H NMR** (400 MHz, DMSO-d_6)

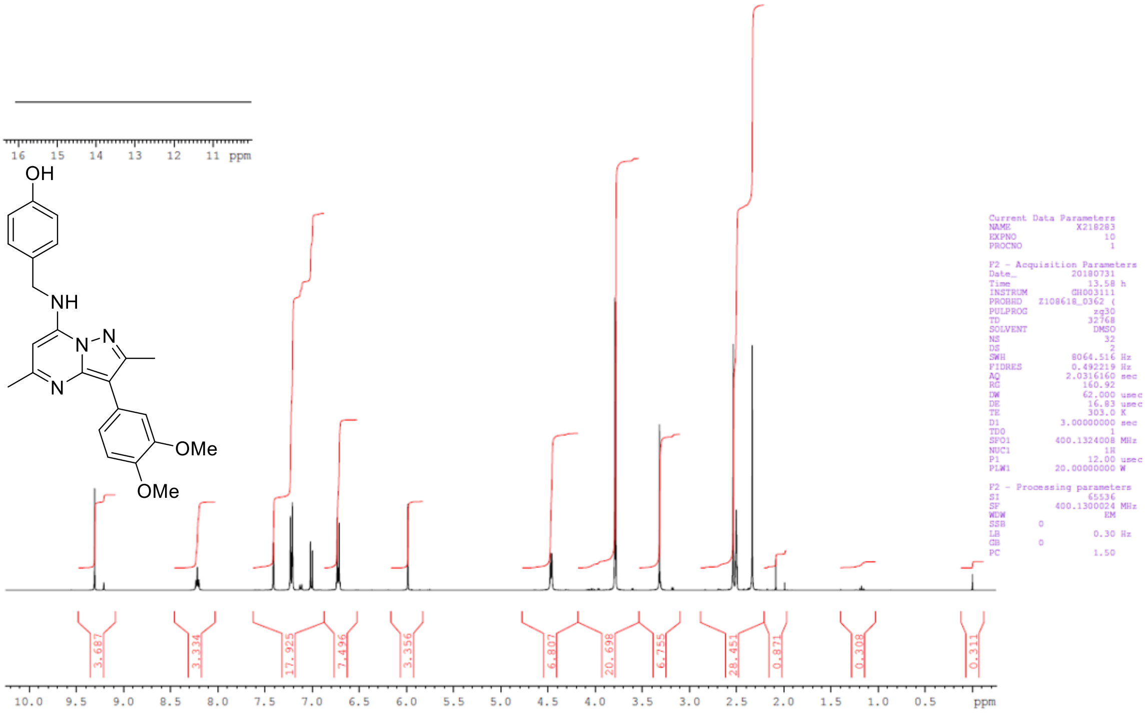

4-(((3-(3,4-Dimethoxyphenyl)-2,5-dimethylpyrazolo[1,5-a]pyrimidin-7-yl)amino)methyl)phenol **7**: **^{13}C NMR** (101 MHz, DMSO-d_6)

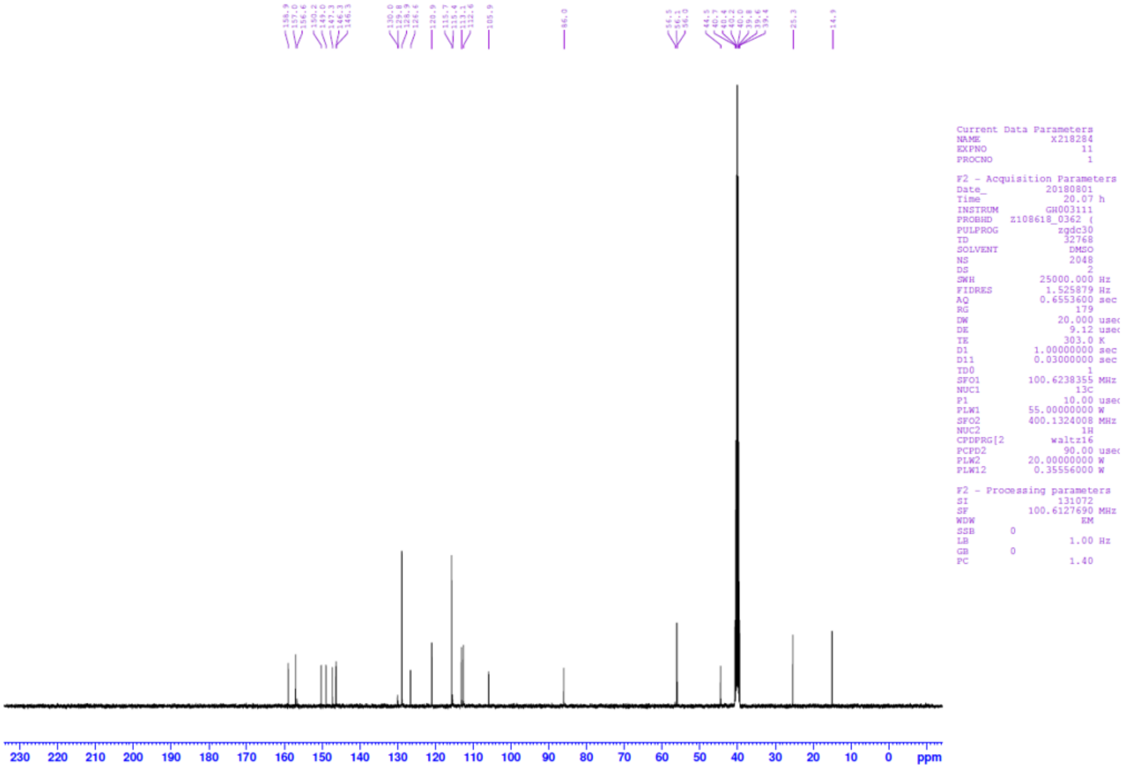

4-(((3-(3,4-Dimethoxyphenyl)-2,5-dimethylpyrazolo[1,5-*a*]pyrimidin-7-yl)amino)methyl)phenyl sulfofluoridate **8**: **^{1}H NMR** (400 MHz, DMSO-d$_6$)

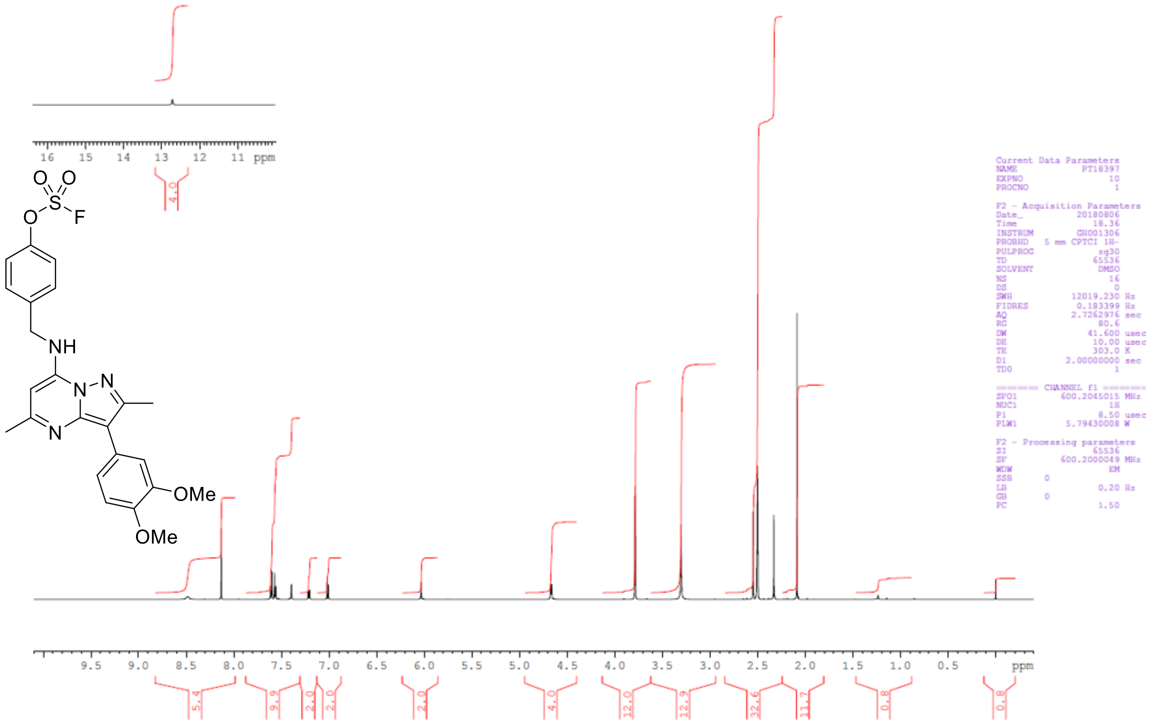

4-(((3-(3,4-Dimethoxyphenyl)-2,5-dimethylpyrazolo[1,5-*a*]pyrimidin-7-yl)amino)methyl)phenyl sulfofluoridate **8**: **^{13}C NMR** (151 MHz, DMSO-d$_6$)

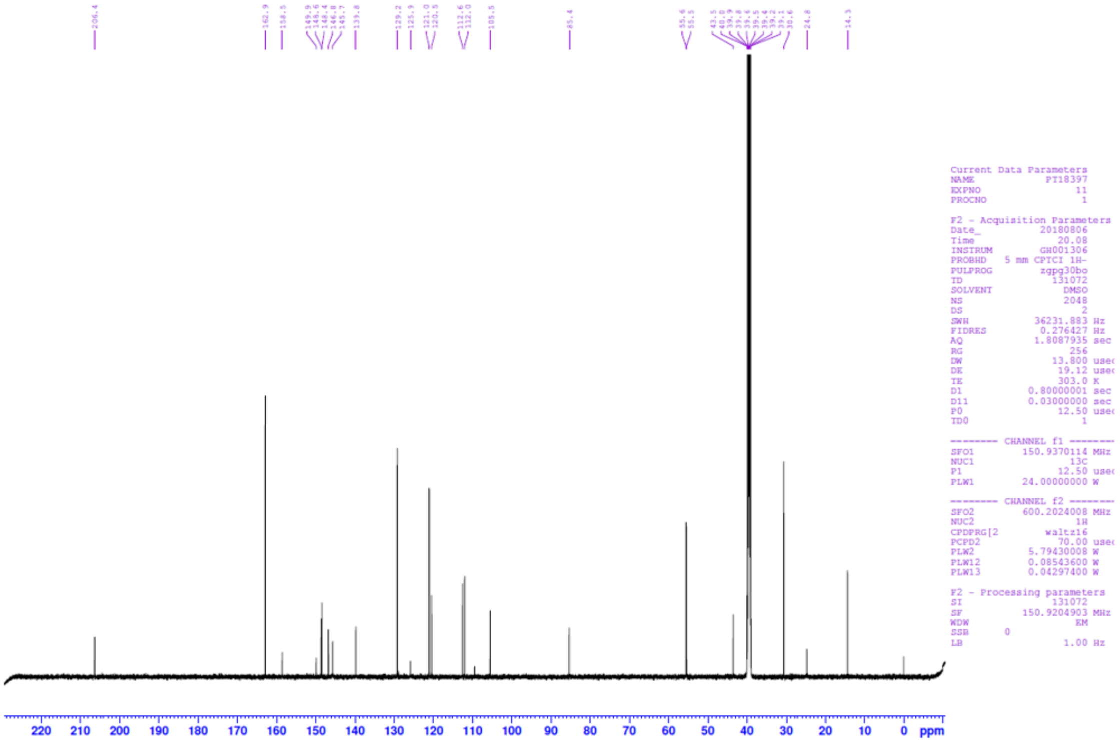

4-(((3-(3,4-Dimethoxyphenyl)-2,5-dimethylpyrazolo[1,5-*a*]pyrimidin-7-yl)amino)methyl)phenyl sulfofluoridate **8**: **^{19}F NMR** (376 MHz, DMSO-d6)

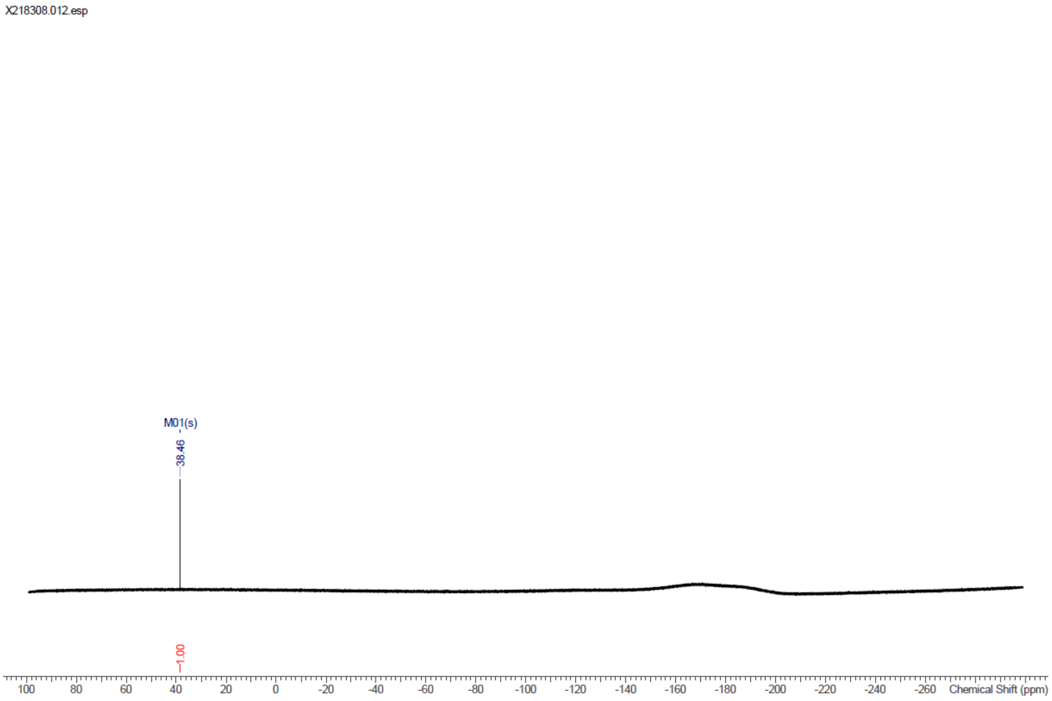

4-(((3-(3,4-Dimethoxyphenyl)-2,5-dimethylpyrazolo[1,5-a]pyrimidin-7-yl)amino)methyl)phenyl methanesulfonate **9**: ^{1}**H NMR** (400 MHz, DMSO-d$_6$)

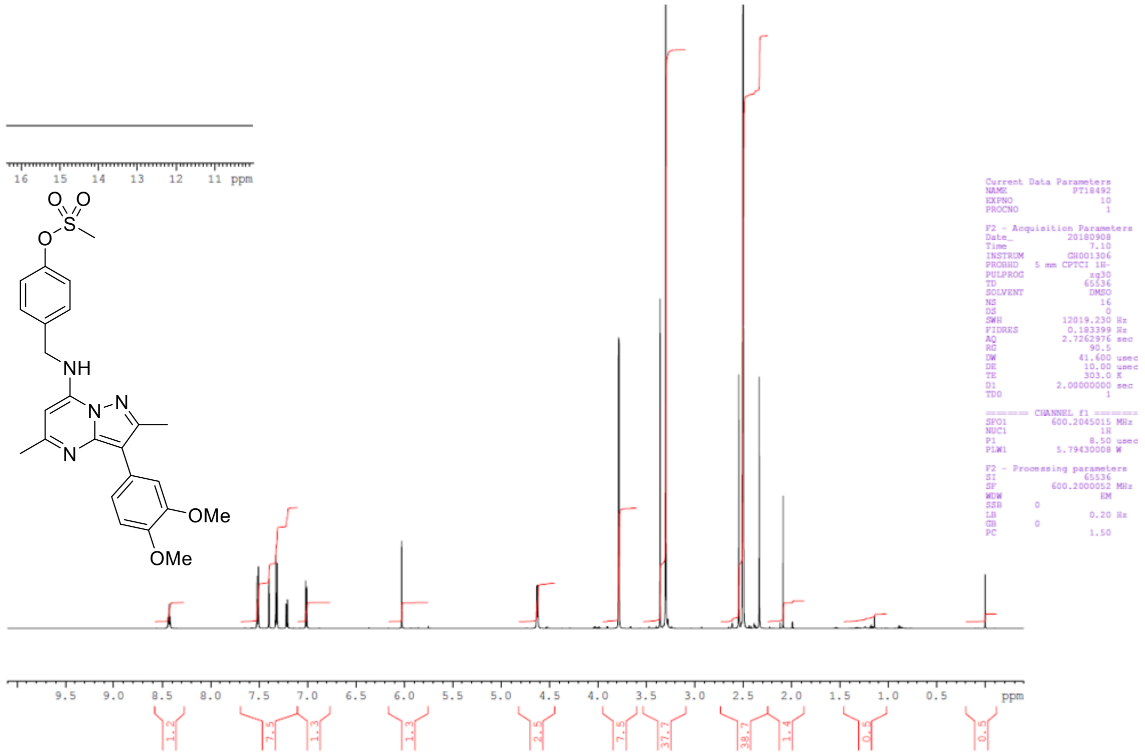

4-(((3-(3,4-Dimethoxyphenyl)-2,5-dimethylpyrazolo[1,5-a]pyrimidin-7-yl)amino)methyl)phenyl methanesulfonate **9**: ^{13}**C NMR** (151 MHz, DMSO-d$_6$)

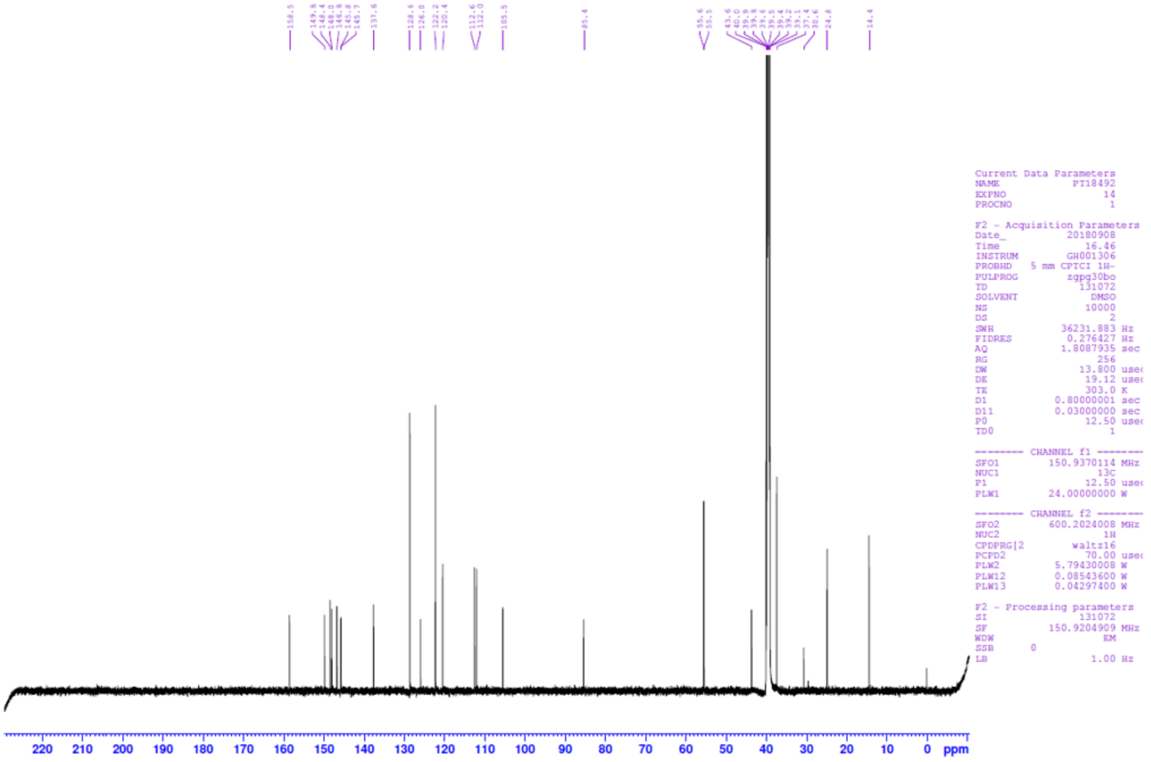

5-(7-((4-Hydroxybenzyl)amino)-2,5-dimethylpyrazolo[1,5-a]pyrimidin-3-yl)-2-methoxyphenol **10**: ^{1}**H NMR** (400 MHz, DMSO-d$_6$)

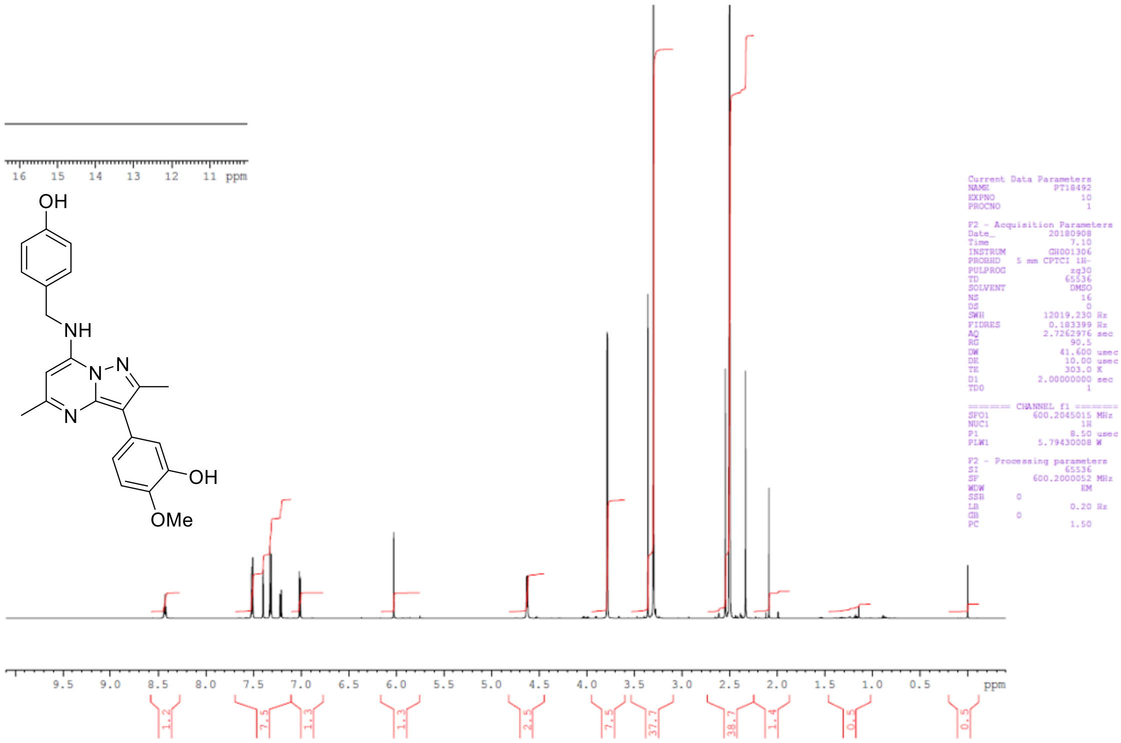

5-(7-((4-Hydroxybenzyl)amino)-2,5-dimethylpyrazolo[1,5-a]pyrimidin-3-yl)-2-methoxyphenol **10**: ^{13}**C NMR** (101 MHz, DMSO-d$_6$)

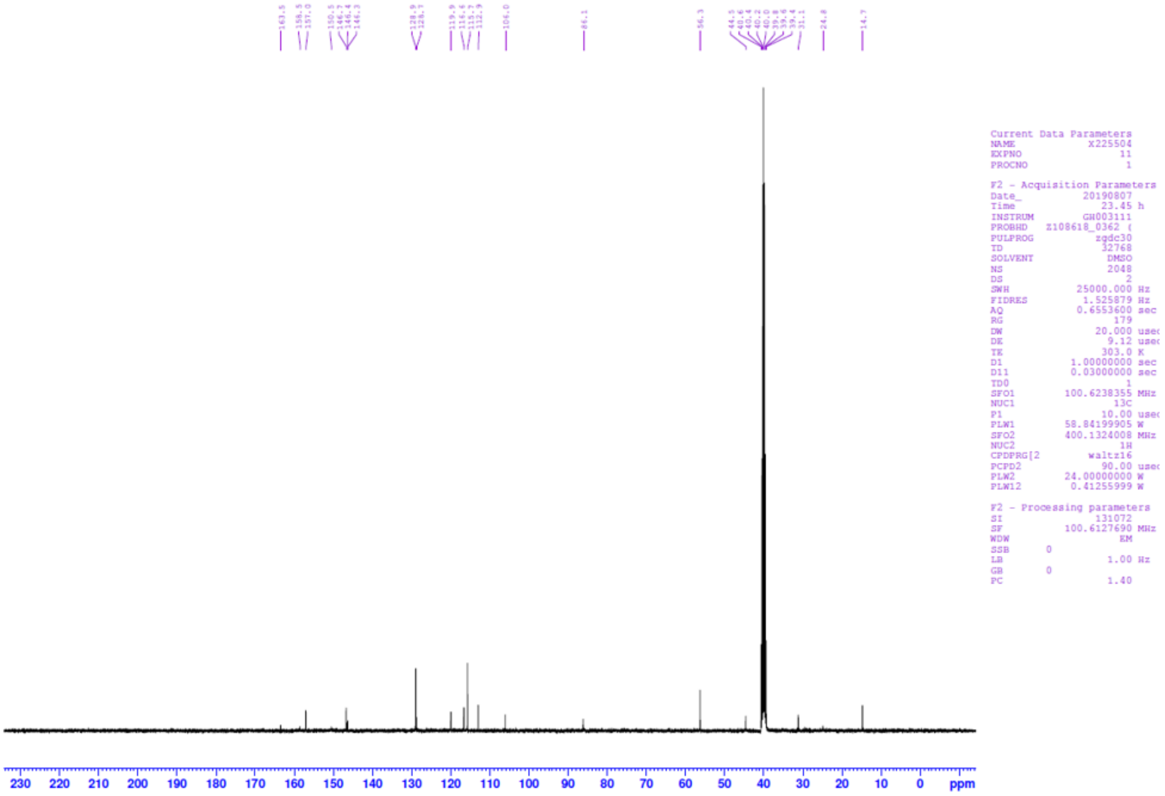

4-(((3-(3-((Fluorosulfonyl)oxy)-4-methoxyphenyl)-2,5-dimethylpyrazolo[1,5-a]pyrimidin-7-yl)amino)methyl)phenyl sulfurofluoridate **11**: **^{1}H NMR** (400 MHz, DMSO-d_6)

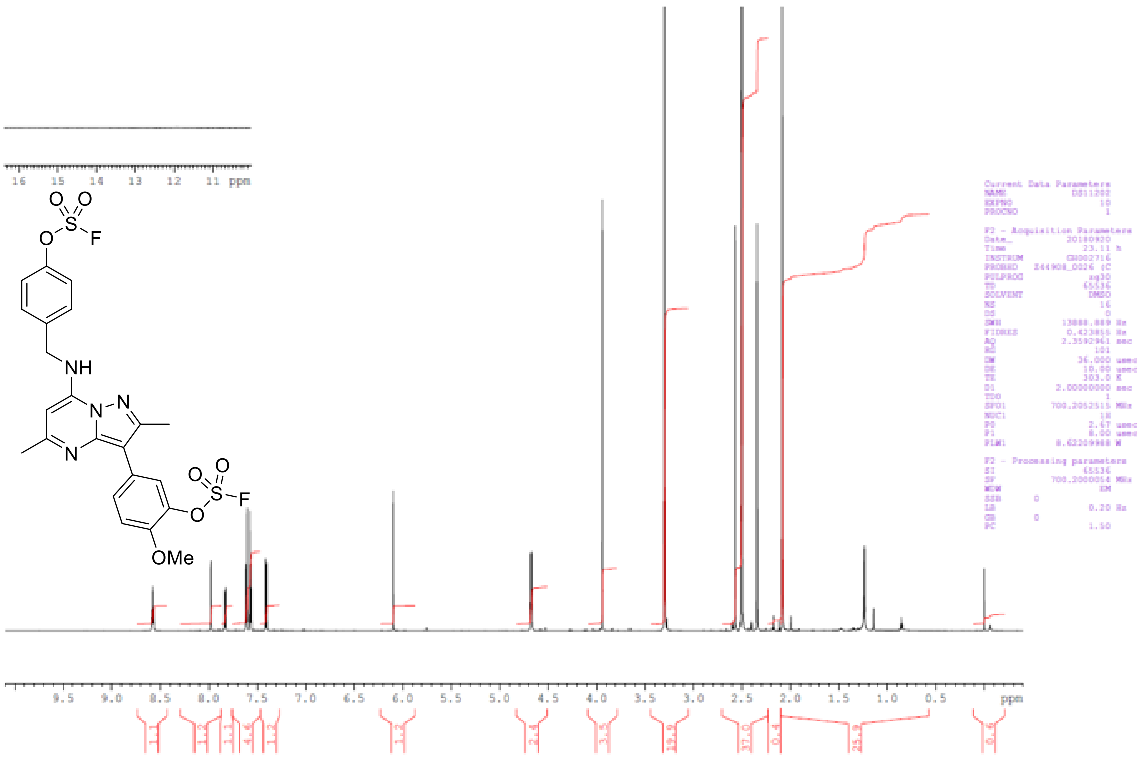

4-(((3-(3-((Fluorosulfonyl)oxy)-4-methoxyphenyl)-2,5-dimethylpyrazolo[1,5-a]pyrimidin-7-yl)amino)methyl)phenyl sulfurofluoridate **11**: **^{13}C NMR** (176 MHz, DMSO-d_6)

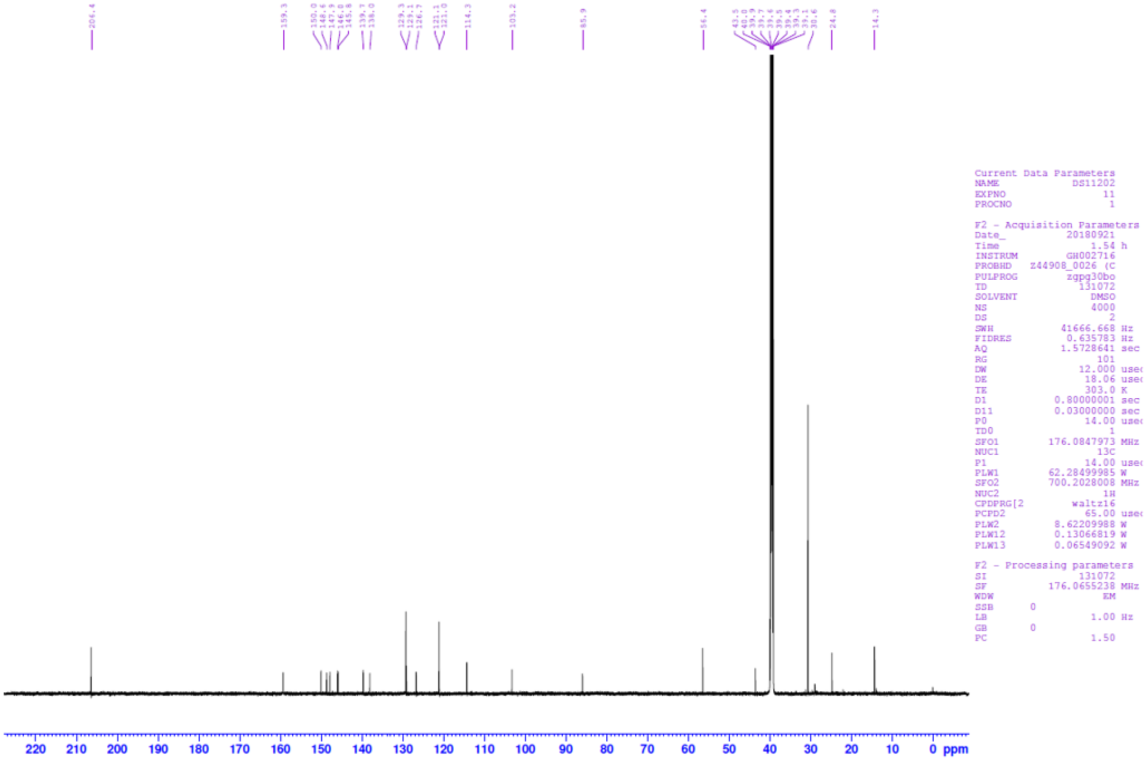

4-(((3-(3-((Fluorosulfonyl)oxy)-4-methoxyphenyl)-2,5-dimethylpyrazolo[1,5-a]pyrimidin-7-yl)amino)methyl)phenyl sulfurofluoridate **11**: **^{19}F NMR** (500 MHz, DMSO-d_6) δ = 40.31 (s, 1F), 38.43 (s, 1F).

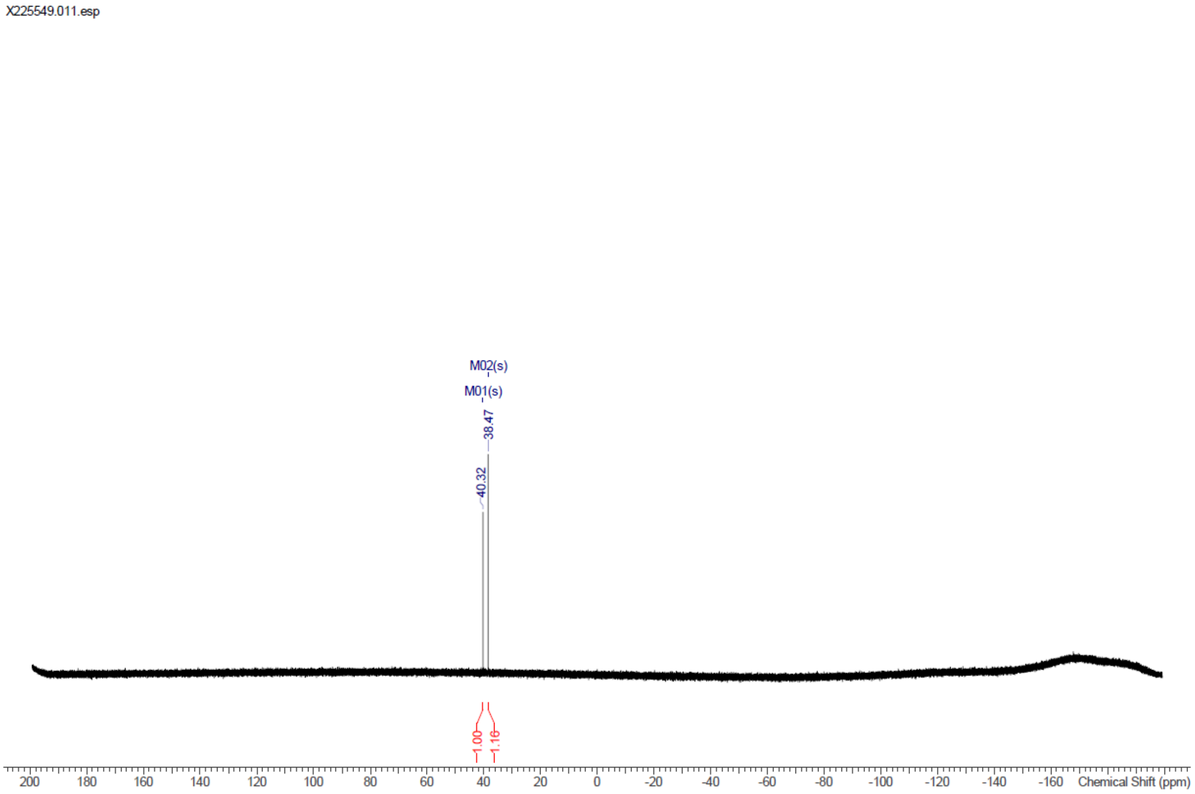

References

1. C. Vonrhein, C. Flensburg, P. Keller, A. Sharff, O. Smart, W. Paciorek, T. Womack and G. Bricogne, *Acta Cryst*., 2011, **D67**, 293–302.

2. I. J. Tickle, C. Flensburg, P. Keller, W. Paciorek, A. Sharff, C. Vonrhein and G. Bricogne, STARANISO. Cambridge, United Kingdom: Global Phasing Ltd.

3. W. Kabsch, *Acta Cryst*., 2010, **D66**, 125–132.

4. P. R. Evans, *Acta Cryst*., 2006, **D62**, 72–82.

5. P. R. Evans and G. N. Murshudov, *Acta Cryst*., 2013, **D69**, 1204–1214.

6. M. D. Winn, C. C. Ballard, K. D. Cowtan, E. J. Dodson, P. Emsley, P. R. Evans, R. M. Keegan, E. B. Krissinel, A. G. W. Leslie, A. McCoy, S. J. McNicholas, G. N. Murshudov, N. S. Pannu, E. A. Potterton, H. R. Powell, R. J. Read, A. Vagin and K. S. Wilson, *Acta. Cryst*., 2011, **D67**, 235–242.

7. G. N. Murshudov, A. A. Vagin and E. J. Dodson, *Acta. Cryst*., 1997, **D53**, 240–255.

8. P. Emsley and K. Cowtan, *Acta Cryst*., 2004, **D60**, 2126–2132.

9. Chemical Computing Group's PSILO software, Version 2022.1207.
